# Marginal effects of public health measures and COVID-19 disease burden in China: A large-scale modelling study

**DOI:** 10.1371/journal.pcbi.1011492

**Published:** 2023-09-18

**Authors:** Zengmiao Wang, Peiyi Wu, Lin Wang, Bingying Li, Yonghong Liu, Yuxi Ge, Ruixue Wang, Ligui Wang, Hua Tan, Chieh-Hsi Wu, Marko Laine, Henrik Salje, Hongbin Song

**Affiliations:** 1 State Key Laboratory of Remote Sensing Science, Center for Global Change and Public Health, Faculty of Geographical Science, Beijing Normal University, Beijing, China; 2 Department of Genetics, University of Cambridge, Cambridge, United Kingdom; 3 Beijing Center for Disease Prevention and Control, Beijing, China; 4 Center of Disease Control and Prevention, PLA, Beijing, China; 5 Translational and Functional Genomics Branch, National Human Genome Research Institute, National Institutes of Health, Bethesda, Maryland, United States of America; 6 Mathematical Sciences, University of Southampton, Southampton, United Kingdom; 7 Finnish Meteorological Institute, Meteorological Research Unit, Helsinki, Finland; Kirby institute, University of New South Wales, AUSTRALIA

## Abstract

China had conducted some of the most stringent public health measures to control the spread of successive SARS-CoV-2 variants. However, the effectiveness of these measures and their impacts on the associated disease burden have rarely been quantitatively assessed at the national level. To address this gap, we developed a stochastic age-stratified metapopulation model that incorporates testing, contact tracing and isolation, based on 419 million travel movements among 366 Chinese cities. The study period for this model began from September 2022. The COVID-19 disease burden was evaluated, considering 8 types of underlying health conditions in the Chinese population. We identified the marginal effects between the testing speed and reduction in the epidemic duration. The findings suggest that assuming a vaccine coverage of 89%, the Omicron-like wave could be suppressed by 3-day interval population-level testing (PLT), while it would become endemic with 4-day interval PLT, and without testing, it would result in an epidemic. PLT conducted every 3 days would not only eliminate infections but also keep hospital bed occupancy at less than 29.46% (95% CI, 22.73–38.68%) of capacity for respiratory illness and ICU bed occupancy at less than 58.94% (95% CI, 45.70–76.90%) during an outbreak. Furthermore, the underlying health conditions would lead to an extra 2.35 (95% CI, 1.89–2.92) million hospital admissions and 0.16 (95% CI, 0.13–0.2) million ICU admissions. Our study provides insights into health preparedness to balance the disease burden and sustainability for a country with a population of billions.

## Author summary

As China continues to be challenged by successive SARS-CoV-2 variants, rethinking population health, broader than health-care system alone, is a prerequisite for health preparedness. China has introduced several changes to its public health measures in response to evolving pandemic risk, however, the effectiveness of public health measures in controlling COVID-19 outbreaks in China and their impacts on the associated disease burden have not been quantitatively assessed at the national level. Here, we developed a stochastic age-stratified metapopulation model based on high resolution human mobility data to address these questions. Our results suggest that there’s marginal effect between the testing speed and reduction in the epidemic duration. Rapid implementation of population-level testing and a short response lag would be critical if aiming at suppression of potential future SARS-CoV-2 waves in China. Furthermore, the underlying health conditions would lead to extra hospital admissions and ICU admissions, where obesity is expected to contribute to most extra hospital admissions, while hypertension could contribute to most extra ICU admissions.

## Introduction

Since the first COVID-19 wave in Wuhan in 2020, China implemented the strict control strategy to combat SARS-CoV-2. This strategy successfully contained hundreds of SARS-CoV-2 outbreaks across the country and was estimated to have averted more than one million deaths in China [1,2]. However, the effectiveness of the strategy appears to have been substantially challenged by the Omicron and other highly transmissible variants. Major Omicron outbreaks occurred in Shanghai, Jilin, and Hong Kong in the spring of 2022 [3–5], and there was a rapid surge of infections across China in December 2022 after the relaxation of the strategy [6–10]. Although WHO declared end to COVID-19’s emergency phase [11], the effectiveness of public health measures against Omicron-like variant is still being debated, especially with regard to the COVID-19 disease burden considering underlying health conditions in the future wave.

**Fig 1:**
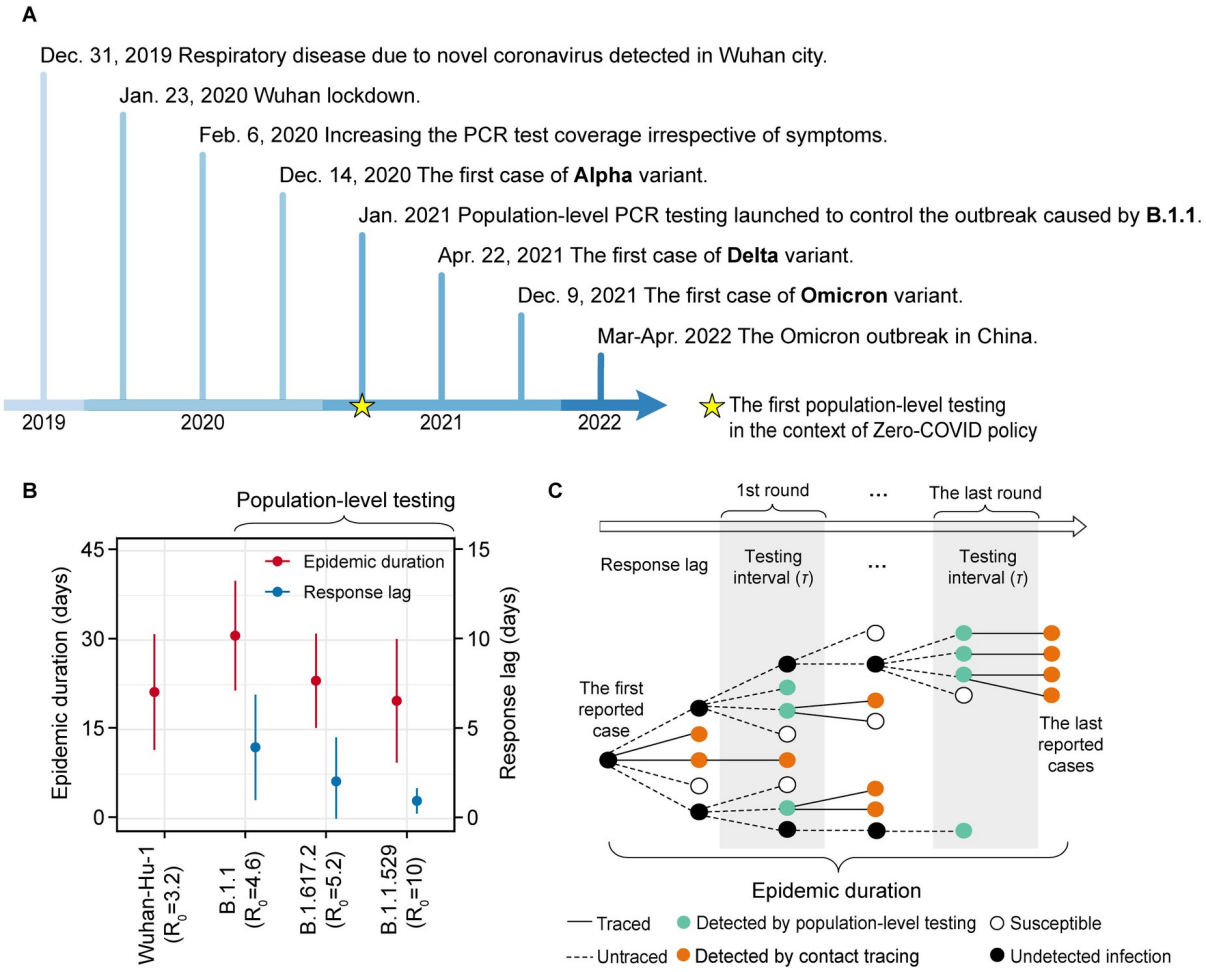
Evolution of the public health measures in China and successfully controlled COVID-19 outbreaks. (**A**) Key changes in the control strategy in China. Initial population-level testing is highlighted with a yellow star, and the first imported case of each variant of concern is in bold. (**B**) Successfully controlled COVID-19 outbreaks, stratified according to the SARS-CoV-2 variant causing each outbreak. From the first outbreak of B.1.1 in January 2021, multiple rounds of population-level testing were introduced. Red error bars correspond to measures of epidemic duration (i.e., time interval from the first to the last reported case in each outbreak). Blue error bars correspond to the response lag (i.e., time interval from the first reported case to the start of population-level testing at city level in each outbreak). The basic reproduction number (*R*_0_) for Wuhan-Hu-1, B.1.1, B.1.617.2 (Delta), and B.1.1.529 (Omicron) was set to be 3.2, 4.6, 5.2 and 10, respectively, according to previous studies [31,79–81]. (**C**) Illustration of population-level testing and contact tracing employed in China. The testing interval is the time to finishing one round of population-level testing.

China’s public health measures mainly includes travel restrictions between cities and testing (with contact tracing and isolation) for almost three years. These measures have been adjusted in response to the changing pandemic risk (Fig 1A). During the first wave in 2020, China banned travel to and from Wuhan and implemented strict social distancing (Fig 1A). In January 2021, China began to adopt multiple rounds of population-level testing (or screening based on the definition of European Centre for Disease Prevention and Control [12]) combined with contact tracing for rapid case identification in response to the increasing risk of imported cases caused by multiple variants of SARS-CoV-2 (Fig 1A). This change represents the key transition from passive surveillance to active surveillance in China. Over the past three years, China has developed a robust PCR testing capacity, with a daily capacity of over 100 million single tube tests, and a potential testing capacity of over one billion people per day if a 1:10 pooled testing strategy is employed [13].

The critical role of testing specific populations in pandemic control, e.g., health workers [14], quarantined individuals [15,16], and international travelers [17], has been demonstrated. Mass testing was also implemented in some countries considering the existence of presymptomatic or asymptomatic transmission [18,19], with diverse purposes (e.g., reducing the SARS-CoV-2 prevalence in Slovakia [20–22], investigating the symptoms of COVID-19 and monitoring SARS-CoV-2 prevalence in England [23,24]). Rapid and/or frequent testing (two or three times per week) has been used to mitigate the transmission of SARS-CoV-2 at large public universities in the United States [25] and in schools and daycare facilities in Germany [26,27]. However, requirements for population-level testing have not yet been well explored in the setting of Omicron-like variant across 366 Chinese cities with human mobility [28].

Meanwhile, the travel restrictions has been extensively evaluated at different scales, from within-country [29–31] to within-continent [32] and globally [33,34]. Although the travel restrictions slowed the epidemic, they failed to stop the spread of the virus nationwide and globally [31,33,35,36]. Additionally, the potential negative effects have caused great concern [37–41], such as the association between lockdown duration and mental health [42,43]. Although the public health measures in China were relaxed to balance the advantages and disadvantages [6–8] when facing Omicron and its subvariants [44–47], few comprehensive quantitative assessments have been conducted to assess the effectiveness of control measures against Omicron variants and the corresponding COVID-19 disease burden, especially considering the underlying health conditions and high resolution human mobility in 366 cities of China [28,48–50].

In this study, we developed a stochastic age-stratified metapopulation model to evaluate COVID-19 disease burden and public health measures for a single wave in China. We explored the feasibility of combating Omicron-like waves without implementing travel restrictions between cities. The disease burden was also accessed by considering 8 types of the underlying health conditions among the population of China. A massive nationwide mobile phone dataset covering 419 million travel movements among 366 Chinese cities before and during the pandemic was incorporated into the model. Our detailed analysis may provide insights for future pandemic preparedness based on self-monitoring and underlying health conditions in China.

## Results

### Population-level testing is critical if aiming at suppressing Omicron, even without travel restrictions between cities

China successfully controlled dozens of sporadic outbreaks caused by different SARS-CoV-2 variants or lineages, including B.1.1 (4 outbreaks), Delta (11 outbreaks) and Omicron (3 outbreaks), with the duration of each outbreak limited to less than a month and the maximum size of each outbreak limited to 2,500 confirmed cases (Fig 1B and S1 Table). Owing to the emergence of highly contagious Omicron variants, China substantially increased the intensity of population-level testing at city level, as measured by the shorter response lag (i.e., time interval from the first reported case to the start of population-level testing at city level in each outbreak) and testing interval (i.e., the time to finishing one round of population-level testing), compared with that for B.1.1 (Wilcoxon test statistic = 2.5, *P* = 0.04). The duration of each outbreak, as measured by the time interval from the first reported case to the last reported case, was also significantly reduced (Wilcoxon test statistics = 3, *P* = 0.06) (Fig 1B and 1C, S1 Table).

To assess the effectiveness of population-level testing at city level in controlling a highly transmissible variant like Omicron BA.1, we first developed a metapopulation model that incorporates the initial public health measures, including travel restrictions between cities and social distancing (a set of measures aiming to reduce the transmission rate, e.g., wearing masks) but without population-level testing, contact tracing and isolation. Our results reveal the difficulty in controlling a variant with similar transmissibility as Omicron using the control measures previously employed to contain the Wuhan-Hu-1 outbreak, if aiming at suppressing Omicron-like wave (S1–S7 Figs, S2 Table).

**Fig 2:**
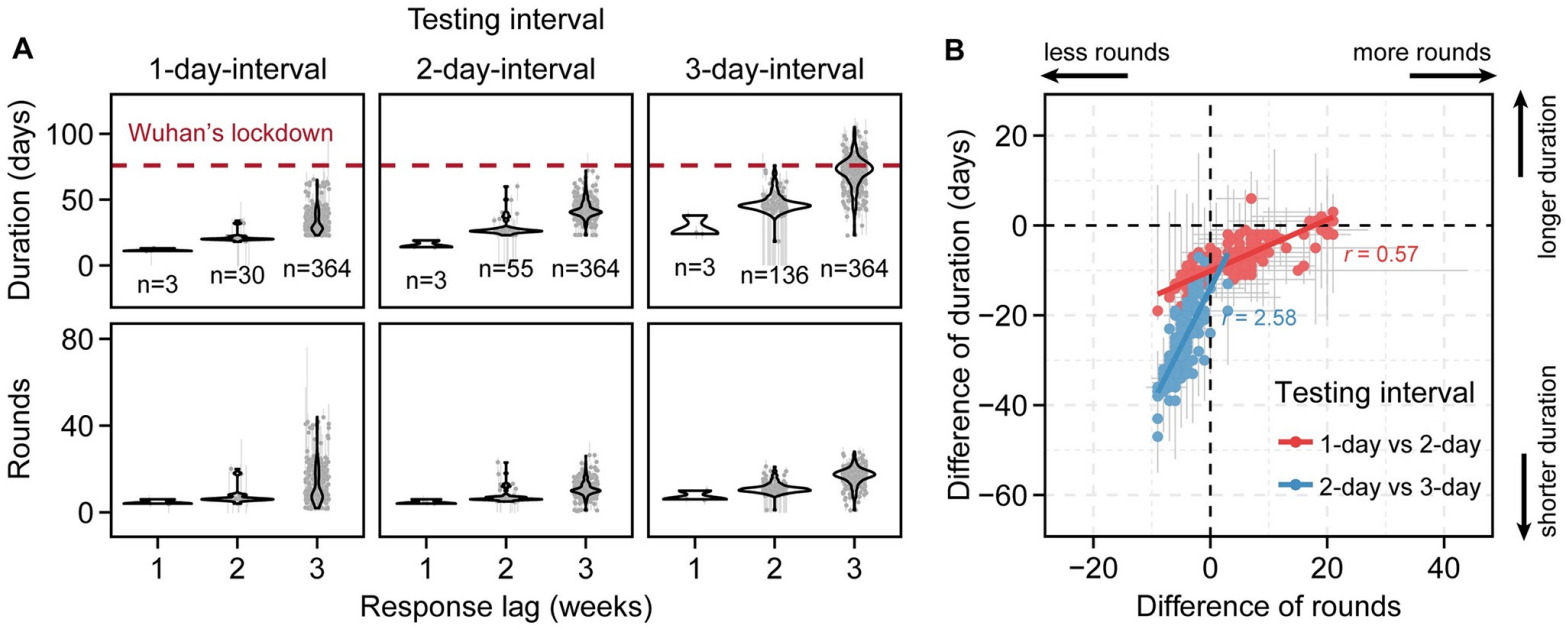
All possible control strategies aiming at suppressing SARS-CoV-2 infections in China during Omicron-like variant wave (*R*_0_ = 10). (**A**) Predicted epidemic duration and rounds of testing across 366 cities in China under different testing intervals and response lags. The dashed red line shows the duration of Wuhan’s lockdown in 2020 (i.e., 76 days) as reference. The number of cities with local outbreaks is labelled below the violin plot. The grey dot represents the median and the grey error bar represents the 95% CI based on the 100 simulation. (**B**) Comparison of epidemic duration and rounds of testing between two successive testing intervals when the response lag was 3 weeks. A negative value corresponds to benefits yielded for duration or rounds by accelerating testing (shortened testing interval), and a positive value corresponds to potential loss for duration or rounds by accelerating testing. The slope for red dots is significantly smaller than that for blue dots (*P* < 0.001). A dot represents a city. The red and blue dots represent median and grey error bar represents the 95% CI based on the 100 simulation. The reduction of social distancing on the transmission rate was set to 18% by considering the effect of only mask wearing against SARS-CoV-2 infection [82]. The vaccine coverage for all age groups was set to 89% (consistent with 86% vaccine coverage in the ≥60 age group by August of 2022 in China) and the effectiveness of China’s inactivated vaccines (BBIBP-CorV and CoronaVac) against infection was set to 40% for Omicron [76,77]. The full list of epidemiological parameters is given in S6 and S7 Tables. The response lag is the time interval from the first reported case to the start of population-level testing at city level in each outbreak. The testing interval is the time to finishing one round of population-level testing.

We then extended the previously developed testing–contact tracing–isolation model [51] into a stochastic age-stratified metapopulation model but without travel restrictions between cities, to assess the controllability of the Omicron-like variant in China. The study period for this model began from September 2022. The sensitivity of PCR tests when administered at different time after infection was also modelled (Section S1.3 of Supplementary Materials). Our results indicate that population-level testing with an interval of ≤ 3 days would be necessary to achieve suppression in an Omicron-like variant wave. Increasing the response lag and testing interval of population-level testing significantly extends the epidemic duration and increases the rounds of testing, resulting in more infections and a higher number of tests (Fig 2A, S8 and S9 Figs; *P* < 0.001 from the *t*-test in S3 and S4 Tables). The strategy with a 3-day testing interval and a response lag of 3 weeks would be least stringent among all possible combinations of control measures aiming at suppressing SARS-CoV-2 infections. Any less-stringent strategy would lead to a failure of suppression for Omicron-like wave [for example, the response lag of 4 weeks or 4-day testing interval could result in whole population isolated in many cities (S5 Table) or longer epidemic duration (S10 Fig)].

We observed a large number of rounds of testing for some cities with a response lag of 3 weeks and a 1-day testing interval (Fig 2A). Although suppression can be achieved in these cities for a short duration, new rounds of population-level testing may be triggered due to the importation of new COVID-19 cases from other cities. By comparison, the effect on rounds of population-level testing together with travel restrictions between cities was also evaluated; no waves were triggered by imported COVID-19 cases (S11 Fig). Sensitivity analysis showed that a SARS-CoV-2 variant with lower basic reproduction number could reduce the demand for testing, but may need fast response if testing interval is 4 days (S12 Fig). We also performed sensitivity analysis by shortening the infectious period (S13 Fig). The outbreak would be harder to control in terms of the number of cities with outbreak and the epidemic duration. Considering the emergence variants with breakthrough (e.g., BQ.1.1 and XBB), we performed sensitivity analysis to assess the feasibility of population-level testing aiming to suppress Omicron-like waves by varying the vaccine effectiveness against infection. The results showed that 3-day testing interval with the response lag of 1 week could control the outbreak even with the vaccine effectiveness against infection of 0 (an extreme situation).

### Marginal effects between decreasing testing interval and reduction in epidemic duration

We observed marginal effects between the testing interval and reduction in the epidemic duration given a response lag of 3 weeks (Fig 2B). Decreasing the testing interval from 3 days to 2 days can largely reduce the rounds of population-level testing and epidemic duration (Fig 2B). However, reducing the testing interval from 2 days to 1 day only gives marginal gain, i.e., the reduction in epidemic duration decreased with a one-round increase in testing (smaller slope for 1-day vs. 2-day testing interval observed with *P* < 0.001 from the *t*-test, compared with that for a 2-day vs. 3-day testing interval). A similar pattern exists under different response lags (S14 Fig). To examine whether the marginal effects between the testing interval and reduction in epidemic duration is caused by the movements of COVID-19 cases among cities, we performed a similar analysis with travel restrictions between cities. The results indicated that the marginal effects persisted (S15 Fig).

### Impact of the public health measures on SARS-CoV-2 Omicron burden in China

According to the above simulations, we assessed the daily hospital beds required and the daily ICU beds required during a highly contagious SARS-CoV-2 variant (like Omicron) outbreak, accounting for hospitalization and ICU admission rates for symptomatic Omicron infection in vaccinated and unvaccinated individuals (S8 Table). The control strategy was set with a 3-day testing interval and response lag of 3 weeks, which is the least stringent strategy among all possible strategies aiming at suppressing SARS-CoV-2 infections based on the simulations. Under this scenario, the average number of SARS-CoV-2 infections per 100 individuals across the cities in China would be 1.34 (95% CI, 1.19–1.50), 1.83 (95% CI, 1.63–2.03), 1.23 (95% CI, 1.10–1.36), and 0.46 (95% CI, 0.41–0.51) for the 0–19, 20–39, 40–59 and ≥ 60 age groups, respectively (S16A Fig), with 34.71 (95% CI, 24.76–48.79) million infections in total.

**Fig 3:**
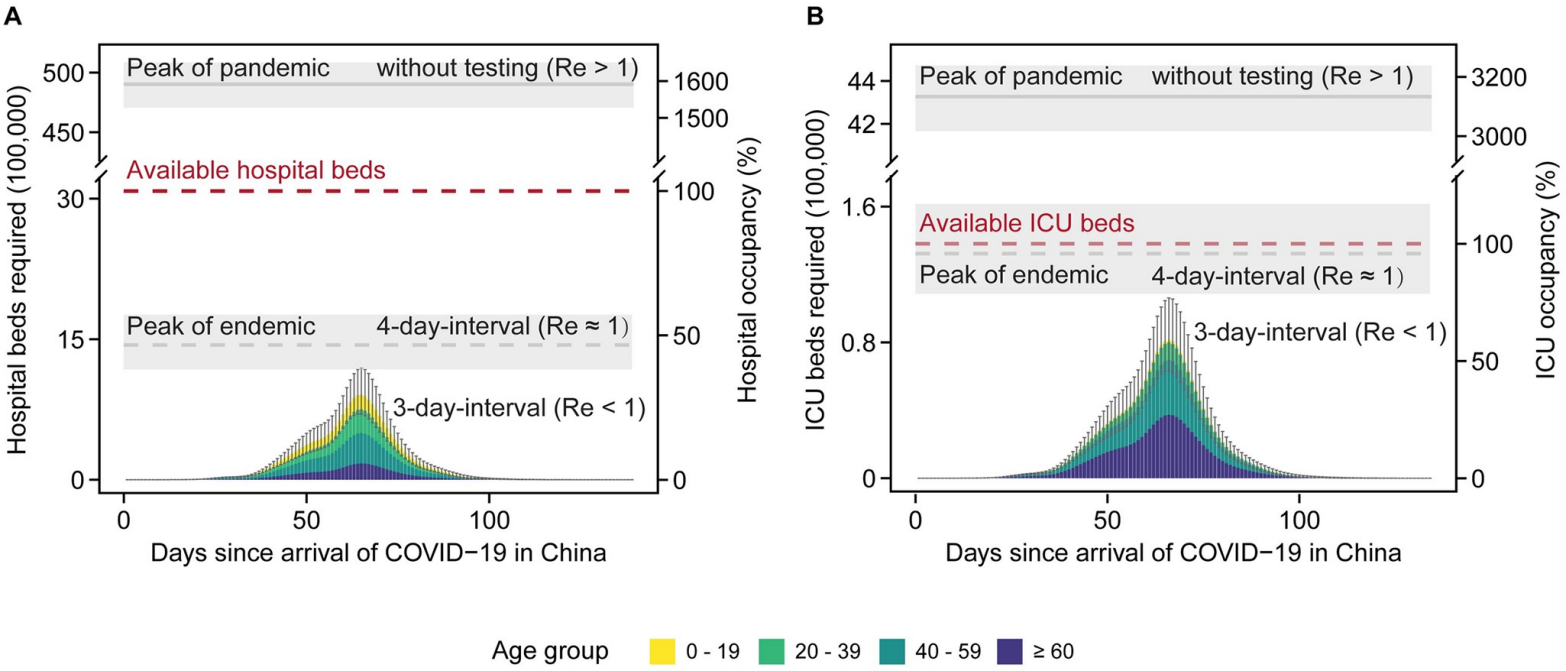
COVID-19 burden for the Omicron-like variant (*R*_0_ = 10) under the control strategy in China. (**A**) Daily required hospital beds for different age groups. (**B**) Daily required ICU beds for different age groups. The control strategy was employed with a testing interval of 3 days and a response lag of 3 weeks (the effective reproduction number *R*_e_ < 1), which is the least stringent strategy aiming at suppressing SARS-CoV-2, as shown in Fig 2A. A strategy with a testing interval of 4 days and a response lag of 3 weeks would lead to the endemic of COVID-19 as shown in the grey dashed line with *R*_e_ ≈ 1. The red dashed line represents the total available hospital beds or ICU beds in China. The grey solid line represents the peak number of required hospital beds or ICU beds during the pandemic without testing (*R*_e_ > 1). The bar represents the median, and the grey error bar or shadow represents the 95% CI for 100 simulations. The vaccine coverage for all age groups was set to be 89%, consistent with 86% vaccine coverage in the ≥60 age group by August of 2022 in China. The response lag is the time interval from the first reported case to the start of population-level testing at city level in each outbreak. The testing interval is the time to finishing one round of population-level testing.

Considering that hospitalization and ICU admission for COVID-19 cases are highly dependent on the underlying health conditions and age, we collected the age-adjusted risk ratios of the hospitalization and ICU admission for 8 types of underlying conditions, hypertension, diabetes mellitus, chronic kidney disease, obesity, chronic obstructive pulmonary disease, asthma, chronic liver disease and cancer, and their corresponding age-specific or region-specific prevalence in the Chinese population. The highest age-adjusted odds ratio was observed in obesity for hospitalization and chronic obstructive pulmonary disease for ICU admission, while hypertension showed the highest prevalence in China (Table 1). Based on these data, the age- and region- specific prevalence and the risk ratios of the underlying health conditions were incorporated to evaluate the COVID-19 burden (Table 1 and S8 Table, see the Methods for more details). During the outbreak projected under this scenario, both the daily required hospital beds and ICU beds for respiratory illness would be under the capacity of the health care system for respiratory illness (3.1 million hospital beds and 138,100 ICU beds in total [50,52,53]), with the highest occupancy of 29.46% (95% CI, 22.73–38.68%) and 58.94% (95% CI, 45.70–76.90%) for hospitalization and ICU beds, respectively (Fig 3). However, without testing, the peak number of daily required hospital beds would be 15.92 (95% CI, 15.28 to 16.51) times the hospital bed capacity in China (Fig 3). Even worse, the peak number of daily required ICU beds would be 31.33 (95% CI, 30.17 to 32.38) times the maximum capacity. We also evaluated the COVID-19 burden under 4-day testing interval. The endemic of COVID-19 could be reached. The required number of hospital beds would still be under capacity, but the ICU capacity may be slightly overwhelmed (Fig 3A and S17 Fig). The results still hold when taking the uncertainty in vaccine effectiveness against hospitalization and ICU admission (S18 and S19 Figs). We also performed the similar analysis based on Hong Kong-specific and Shanghai-specific age-dependent hospitalization and ICU admission rates. The COVID-19 burden would be under the capacity for 4-day testing interval (S20 and S21 Figs), demonstrating the variations in hospitalization and ICU admission rates in China.

**Table 1:** Age-adjusted odds ratios (OR) for hospitalization and ICU admission of infected individuals with underlying health conditions and the prevalence of underlying health conditions in the Chinese population.

Underlying health conditions	OR for hospitalization	OR for ICU admission	Overall prevalence in China
Hypertension	2.01 [83]	2.95 [84]	0.28^c^ [85]
Diabetes mellitus	2.27 [86]	3.07 [84]	0.12^c^ [87]
Chronic kidney disease	3.4 [86]	5.32 [84]	0.11^a^ [88]
Obesity	5.82* [86]	1.43 [89]	0.05^c^ [90,91]
Chronic obstructive pulmonary disease	1.07 [92]	6.66 [84]	0.03^b^ [93]
Asthma	3.83 [86]	2.63 [86]	0.01^c^ [94–96]
Chronic liver disease	2.77 [86]	1.1 [86]	0.007^c^ [97,98]
Cancer	2.88 [86]	0.91 [86]	0.002^b^ [99]

*^:^ hospitalizations for class III obesity

The prevalence data is an average across different groups, either age-specific^a^, region-specific^b^ or both^c^.

Note: Because of the low prevalence of underlying health conditions, risk ratio was approximated by age-adjusted OR in disease burden calculation.

In terms of the peak of isolated population during the outbreak, the cities with smaller population sizes and less movement, may suffer a higher proportion of isolated people than the cities with higher populations under the control strategy with a 3-day testing interval and response lag of 3 weeks (S16B Fig). The "isolated" individuals include the individuals exposed to the SARS-CoV-2 and the infected individuals. We recognize that our study did not distinguish between isolation and quarantine, as we concentrated on the percentage of individuals who were temporarily restricted in their movements. Contact tracing in the cities with small population sizes would be another type of "lockdown", but only a few cities would experience this. The short response lag can substantially reduce the isolated population (S22 Fig), indicating the importance of fast response in cities with small populations.

### The contributions of underlying health conditions

**Fig 4:**
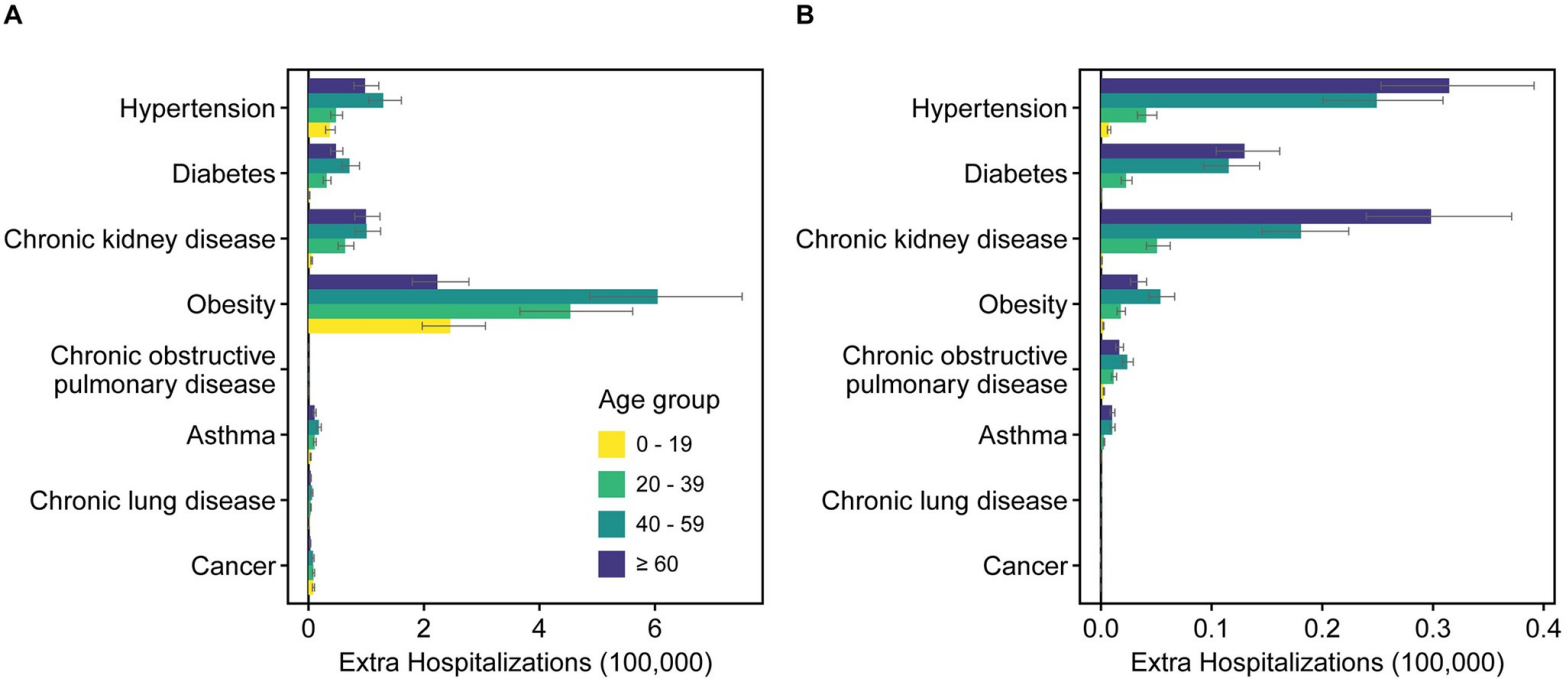
The COVID-19 burden contributed by the underlying health conditions under the control strategy for Omicron-like variant (*R*_0_ = 10) in China. (**A**) The extra number of hospitalizations due to the underlying health conditions for different age groups. (**B**) The extra number of ICU admissions due to underlying health conditions for different age groups. The bar denotes median, and the grey error bar represents the 95% CI for 100 simulations. The extra number of hospitalizations/ICU admissions is the difference between the hospitalizations/ICU admissions considering the underlying health conditions and the hospitalizations/ICU admissions without considering the underlying health conditions. The control strategy was employed with 3 days of testing interval and 3 weeks of response lag (i.e., the least stringent control strategy). The response lag is the time interval from the first reported case to the start of population-level testing at city level in each outbreak. The testing interval is the time to finishing one round of population-level testing.

Projected by the age-stratified metapopulation model under the least stringent control strategy, 1.33 (95% CI, 1.07–1.65) million hospital admissions and 0.10 (95% CI, 0.08–0.12) million ICU admissions in total were estimated without considering the underlying health conditions (S23 Fig). After calibration for the underlying conditions (Table 1 and S8 Table, see the Supplementary Materials for more details), an extra 2.35 (95% CI, 1.89–2.92) million hospital admissions and 0.16 (95% CI, 0.13–0.2) million ICU admissions were estimated under the least stringent control strategy, corresponding to a 1.77-fold (95% CI, 1.15–2.72) and 1.67-fold (95% CI, 1.08–2.57) of that under the scenario without underlying conditions, respectively. The 40–60 age group would account for 39.96% of extra hospital admissions and the ≥ 60 age group would account for 50.30% of extra ICU admissions (Fig 4). Among the underlying health conditions, obesity could contribute to most extra hospital admissions (Fig 4A), while hypertension is expected to contribute to most extra ICU admissions (Fig 4B). Interestingly, for the 0–19 age group, obesity would account for the most extra hospital admissions among all underlying health conditions.

## Discussion

China was one of the first countries to adopt the strict control strategy to control SARS-CoV-2 outbreaks in 2020. Recent adjustment of COVID-19 responses (i.e., "20 measures" and "10 new measures") indicated the end of this strategy [6,7]. However, comprehensive quantitative assessments of the public health measures and the corresponding COVID-19 disease burden in 366 Chinese cities is still lacking. We developed a stochastic age-stratified metapopulation model to address these questions. The modelling framework allows us to assess specific implementation strategies in terms of epidemic duration, rounds of population-level testing, and public health burdens of COVID-19 considering the underlying health conditions and the high-resolution human mobility in 366 cities of China.

Our results indicate that the rapid implementation of population-level testing with 3-day testing interval and a response lag ≤ 3 weeks would be critical if aiming at suppression of potential future SARS-CoV-2 waves in China. Increasing the testing interval to 4 days would lead to endemic of COVID-19 and result in slightly overwhelming the ICU capacity. Compared with previous studies [28,48–50], the outcomes in our study is more actionable. The reported serial interval for Omicron is between 2 and 4 days [54–56]. The sensitivity analysis by lowering basic reproduction number and shortening the infectious period indicated that the serial interval/generation time, the basic reproduction number and the response lag would have impact on the testing interval required for controlling outbreaks, but the generation time/serial interval should contribute most. Considering the fast evolution of SARS-CoV-2 and the importance of the generation time/serial interval [57], these key epidemiological parameters should be monitored continually [58], by contact tracing [59,60] or sequencing data [61].

Given the negative effects of lockdown on lifestyle and mental health [38,39], population-level testing has been proposed as a critical component of the public health measures in China, with relatively minor disruption to daily life. Testing capacity may be an issue for low- to middle- income regions. Rapid antigen testing may be an alternative solution [62], with high frequency and short turnaround time to compensate for the lower sensitivity compared with PCR tests [63–65]. A previous study showed that home antigen test sensitivity was 64% compared with same-day RT-PCR [66]. If 3-day interval is applied to PCR test, 2-day interval [1/ (3*0.64) = 0.52, that is about 2-day interval] would have to be used for rapid antigen test. Optimal pooled testing strategies can also be used in regions with limited resources [67,68]. Additionally, various rapid methods of screening and diagnosis of SARS-CoV-2 have recently been developed, for example, the Lolli-method (effective high-throughput RT–qPCR [26]), ADESSO (Accurate Detection of Evolving SARS-CoV-2 through SHERLOCK (Specific High Sensitivity Enzymatic Reporter UnLOCKing) Optimization, results within 1 hour, with sensitivity and specificity comparable to RT–qPCR and less than 5€ per test [69]), OPTIMA-dx (a sensitive, robust, rapid, one-pot assay [70]), and DNA aptamer-conjugated graphene field-effect transistor (GFET) biosensor platform (label-free, results within 20 minutes, conducted with an ultrasensitive handheld wireless readout device [71]). Traveler screening and wastewater surveillance may facilitate the early detection of outbreaks and would likely reduce the amount of population-level testing required. Another important finding is the marginal effects between the testing interval and the benefit of a reduction in epidemic duration. Shortening the testing interval can significantly reduce the epidemic duration. However, this benefit is diminished at a 1-day testing interval. Previous studies showed that high percentage of contacts successfully traced is needed to control the outbreaks with historical variant [72,73]. Population-level testing (essentially another form of contact tracing) was used to compensate for the low percentage of contacts successfully traced (usually about 17%) in practice.

The use of real-time human mobility data presents an important opportunity to understand the dynamics and control of SARS-CoV-2 in a large country with modern transport systems within a densely populated setting. To quantify the potential severity of the Omicron outbreak, we used a counterfactual assumption that between-city travel restrictions would not be implemented. Our results demonstrate that the effectiveness of travel restrictions depends upon both the timing of implementation and the specific variant. With timely travel restrictions between cities, the number of cities with outbreaks can be significantly reduced. Owing to the economic and social stresses imposed by this type of intervention, trade-offs between economic and public health objectives should be considered.

With the age-stratified metapopulation model incorporating population-level testing and contact tracing, the COVID-19 burden was also evaluated. Due to mass vaccination (especially in the ≥ 60 age group with 86% vaccine coverage by August 2022 in China), the hospital admissions and ICU admissions would be largely reduced. However, the disease burden would still overwhelm the health care system for respiratory illness without the testing. The underlying health conditions would increase the requirements for hospital and ICU beds. After calibrating the risk ratios and the prevalence of underlying health conditions, the 40–60 and ≥ 60 age groups would contribute to most extra hospital admission and ICU admission respectively, if the least stringent control strategy were employed. Among the underlying health conditions, obesity would contribute to most extra hospital admissions, while the hypertension could contribute to most extra ICU admissions. Interestingly, for 0–19 age group, obesity is expected to account for most extra hospital admissions. This is due to the relatively high prevalence of obesity (2.3%) in the young population and high hospitalization rate. More attention should be given to children and adolescents in regions with an increasing prevalence of obesity. We note that these estimations are based on the least-stringent control strategy. In our analysis, the independence among different underlying conditions is assumed. The impacts of multiple comorbid conditions for the same individuals on COVID-19 burden should be investigated, particularly in regions with an ageing population. Due to the scarcity of data, we also assumed that the age distribution of the prevalence of underlying health conditions is consistent between cities and country. The age-adjusted odds ratios (OR) for hospitalization and ICU admission for 8 types of underlying conditions were collected from multiple sources. Incomparability may exist among them. In addition, the hospitalization and ICU admission rate should also be calibrated to account for the underlying health conditions in a detailed way in the future.

There are limitations to this study. Under-reporting may have existed during the initial wave of COVID-19 in 2020 in China. Although different detection rates for symptomatic individuals were modeled, regional variations might not have been fully captured during that period. Additionally, human mobility is often influenced by holidays and other factors, which could impact the spread of the virus. While a typical outbreak scenario was simulated in the study, it is essential to acknowledge that real-world situations might be more complex and dynamic. Future work could focus on the changing epidemiological parameters of new variants [58]. Model calibration should be performed if vaccine effectiveness against infection with emerging variants is maintained, especially for the difference between breakthrough infections in vaccinated populations and waning vaccine effectiveness.

To conclude, we found that the epidemic duration of COVID-19 in China over the past 2 years was reduced by population-level testing. Marginal effects between the testing interval and the reduction in epidemic duration was observed. The control strategy with frequent population-wide testing would keep the COVID-19 burden under a manageable scale in China. The population-level testing might balance disease control and the harm to health, economies, and societies caused by travel restrictions between cities. We hope that the lessons learned from the public health measures in China may help to inform the health preparedness of COVID-19 pandemic, especially considering the unpredictable evolution of SARS-CoV-2 and the high prevalence of underlying conditions.

## Methods

### Epidemiological data

We collected daily official case reports from the health commission websites in 34 provincial-level administrative units and 366 city-level units [31]. Demographic data for each city were collected from the China City Statistical Yearbook 2019 (http://olap.epsnet.com.cn/).

### Human mobility data

Human movement in China can be observed directly using mobile phone data with Baidu location-based services. We obtained both the recorded movements and relative volume of inflows, outflows, and internal movement among cities (n = 366) from the migration flows database (http://qianxi.baidu.com/) from 1 January 2019 to 1 April 2020. We considered the averaged between-city travel flow in 2019 as the flow at baseline and constructed a flow network across 366 cities in China to simulate SARS-CoV-2 transmission across Chinese cities. The travel flow during the first COVID-19 wave in 2020 was used to evaluate the effectiveness of initial version of public health measures.

### Metapopulation models

To evaluate the effectiveness of the public health measures in China and incorporate variations in nonpharmaceutical interventions (NPIs), we developed three metapopulation models with increasing complexity. We first introduced the baseline metapopulation model to set up the context of modelling. Then, we extended the baseline model to account for social distancing to evaluate NPIs during the first epidemic wave and the Omicron-like wave in China. Finally, we developed a stochastic age-stratified metapopulation model that included population-level testing and contact tracing based on previous well-established models [74,75] to illustrate the effectiveness of the control strategy in a highly transmissible variant waves (e.g., Omicron BA.1) and quantitatively assess the number of infections and disease burden in each age group. The initial condition of the simulation is set as September 2022 since we performed the modeling analysis based on the vaccine coverage by August of 2022 in China. Please see the Supplementary Materials for more details.

### Simulation scenarios

According to the fitted metapopulation model with social distancing and daily case reports from each city in the first wave of COVID-19, we simulated different scenarios by varying the strength of social distancing, travel restrictions between cities, vaccine coverage, and SARS-CoV-2 variants. The effectiveness of China’s two-dose inactivated vaccines (BBIBP-CorV and CoronaVac) against infection was set to 40% for Omicron [76,77] and 59% for Wuhan-Hu-1 [78]. All scenarios used the same epidemic trajectories among cities. Using the age-stratified metapopulation model with population-level testing and contact tracing, we also performed sensitivity analysis for the testing interval and response lag for the variant with the basic reproduction number of 10 and 8. In this age-stratified model, the population-level testing at city level would be launched at response lag of 1, 2, 3, or 4 weeks after the first case was identified in the city. Contact tracing would be trigged for each infection irrespective of the presence of symptoms. The infections and the traced contacts would be isolated. Please refer to the Supplementary Materials.

### The collection of risk ratios of hospitalization and ICU admission for COVID-19 cases with underlying conditions

We searched PubMed for articles reporting the odds ratios of hospitalization and ICU admission for COVID-19 cases with underlying health conditions. The search terms "COV" AND "underlying condition" AND "odds ratio" and "COV" AND "underlying condition" AND "OR" yielded 93 and 3,081 studies, respectively. As the odds ratios are highly dependent on age, the age-adjusted odds ratios are of interest and were collected. After screening, 12 studies remained, and they involved 14 types of underlying health conditions. For each underlying condition, the age-adjusted odds ratios for both hospitalization and ICU admission were needed. After this filter, 11 types of underlying health conditions remained. If multiple studies reported age-adjusted odds ratios, the highest one was selected.

### The prevalence of the underlying health conditions in the Chinese population

For each underlying condition from the above risk ratio collection, we searched the reported prevalence in the Chinese population using PubMed and CNKI. In PubMed, the search terms were "China" AND "prevalence" AND name of the underlying condition AND province. The names of the underlying conditions were hypertension, diabetes, obesity, COPD, liver disease, kidney disease, asthma, cancer, cardiovascular disease, cardiac disorder and chronic respiratory disease. These searches generated 22,333 studies in total. For CNKI, the search terms were the same but in Chinese. This search yielded 3,821 studies. After screening the search results from PubMed and CNKI, most studies were found to be irrelevant. The studies reporting age-specific or province-specific prevalence with the latest publication date were selected. Finally, the prevalence for 8 types of underlying health conditions was collected. The age-adjusted risk ratio and the prevalence are shown in Table 1.

### The calculation of extra COVID-19 burden due to the underlying conditions

To evaluate the disease burden of the Omicron variant, the age-specific number of hospitalizations, and ICU admissions were measured quantitatively based on age-specific hospitalization and ICU admission rates (S8 Table). Previous studies suggested that individuals with underlying health conditions have higher risks of hospitalization and ICU admission. We first calculated the number of hospitalizations and ICU admissions as baseline by assuming all individuals were healthy (i.e., without underlying health conditions), and then calculated the number of hospitalizations and ICU admissions considering underlying health conditions across age groups. Finally, we used the difference between them as an extra disease burden due to underlying health conditions. The number of cases in each age group was derived from the age-stratified metapopulation model with population-level testing and contact tracing. Please see the Supplementary Materials for more details.

## Supporting information

S1 Text Supplementary text. (DOCX)

S1 Fig Heterogeneities in population distribution, intra-city movement reduction, movement inflow reduction, movement outflow reduction, before and after the travel restriction among Chinese cities during the first wave. The shading from light to dark represents the value from low to high. The base layer of the map is provided by GADM (File link: https://gadm.org/download_country.html; License information: https://gadm.org/license.html).
(DOCX)

S2 Fig Travel movements in China before and after the travel restriction. (**A**) Movement inflows in 2019 and 2020, averaged across 366 Chinese cities. The median (solid line) and interquartile range (shading) of values among cities are shown. The vertical dotted line represents the beginning of the Wuhan lockdown on 23rd January 2020. (**B**) Intra-city movements in China before and after travel restriction during the SARS-CoV-2 pandemic between January 1 and March 6, 2020. Line shade indicates the average number of daily travel movements (>300 are shown) for each pair of location. Point size represents the volume of travel inflow.
(DOCX)

S3 Fig Travel movements and transmission pattern of the first SARS-CoV-2 wave in China. (**A**) Travel matrices before and after travel restriction. Lower-triangular values represent travel movements from city *i* to city *j*, and upper-triangular values from city *j* to city *i*. The shading from light to dark represents the volume of travel movements between pairs of cities from low to high. Black box represents the travel movements between cities within a province. The upper panel represents the total movements for each city, inflow (red) and outflow (blue). (**B**) Correlation of time series of reported cases between cities in the first wave of China. Cities are categorized by province and ranked from North (top) to South (bottom).
(DOCX)

S4 Fig Pairwise correlation in daily COVID-19 cases between cities during the first wave in China. (**A**) Each point represents a pair of cities. Synchrony of the epidemics in the two cities is measured by the correlation between the number of cases reported in two cities on each day, using a spatial non-parametric correlation function (black line = estimated curve, dark grey area 95% confidence band). The synchrony declines with increasing geographical distance. (**B**) Pairs of cities are ranked according to the level of travel movements between them (from low to high). The city pairs are then classified into ten categories corresponding to quantiles of the rank. Box and whisker plots show distribution of epidemic synchrony scores for cities in each of the ten categories; The first box represents the correlation among Q1-Q1 pairs, the second is among Q1-Q2 pairs, up to the final bar which is among Q4-Q4 pairs. Pairs of cities with more travel movements have more synchronized epidemics.
(DOCX)

S5 Fig Fits of the meta-population model with the social distancing on transmission rate during the first wave in China. Correlation between the number of cases reported in each city and model fitting by March 6, 2020. Circle size is proportional to the correlation coefficient between time series of reported cases and model prediction for each city.
(DOCX)

S6 Fig Fits of meta-population model with the social distancing on transmission rate to time series of reported cases from cities during the first wave (city with more than 50 cases are shown). The numbers of confirmed cases reported (points) and estimated (lines) each day in each city. Grey areas correspond to pointwise 95% prediction envelopes.
(DOCX)

S7 Fig Effectiveness of public health measures without population-level testing during Wuhan-Hu-1 and Omicron waves in China. (**A**) Predicted dependence of the average epidemic duration across all cities on travel restrictions between cities (from 100% strict restriction to 0% no restriction) and social distancing (a set of measures aiming at reduction of transmission rate, e.g., mask wearing, from strong [100%] to weak [0%]) for Wuhan-Hu-1 variant in the absence of vaccination. The estimated intensity of social distancing on reduction of transmission rate during the first wave in 2020 in China is indicated by the red cross. The fitting performance for the first wave can be found in S5 and S6 Figs. The simulation is based on the specific NPIs implemented during the first wave in 2020, except the strength of travel restrictions between cities. (**B**) Same as in (A) but for Omicron variant. (**C**) Predicted dependence of average epidemic duration across all Chinese cities on vaccine coverage and social distancing for the Omicron variant. The simulation is based on the specific NPIs implemented during the first wave in 2020. In (A) to (C), the Zero-COVID line (i.e., controlling the outbreak within 76 days) is shown as a dashed white line and solid line for Wuhan-Hu-1 and Omicron variants, respectively (see Methods). Note that the darker color with longer duration indicates that the whole population was infected, and the epidemic curve is either thin or flat. Effectiveness of China’s inactivated vaccines (BBIBP-CorV and CoronaVac) against infection was set to 40% for Omicron and 59% for Wuhan-Hu-1. The full list of epidemiological parameters is given in S6 Table.
(DOCX)

S8 Fig Number of COVID-19 cases under different response lags and testing intervals of population-level testing (*R*_0_ = 10). Predicted number of COVID-19 cases across 366 cities in China under different testing intervals and response lags. The grey dot represents the median and the grey error bar represents the 95% CI based on the 100 simulations. The reduction of social distancing on transmission rate is set to 18% by considering the effect of only mask wearing against SARS-CoV-2 infection [8]. The vaccine coverage is set to 89% (consistent with 86% vaccine coverage in the ≥60 age group by August of 2022 in China) and the effectiveness of China’s inactivated vaccine (BBIBP-CorV and CoronaVac) against infection was set to be 40% for Omicron [9].
(DOCX)

S9 Fig Number of tests under different response lags and testing intervals of population-level testing (*R*_0_ = 10). Predicted number of tests across 366 cities in China under different testing intervals and response lags. The grey dot represents the median and the grey error bar represents the 95% CI based on the 100 simulations. The reduction of social distancing on transmission rate is set to 18% by considering the effect of only mask wearing against SARS-CoV-2 infection [8]. The vaccine coverage is set to 89% (consistent with 86% vaccine coverage in the ≥60 age group by August of 2022 in China) and the effectiveness of China’s inactivated vaccine (BBIBP-CorV and CoronaVac) against infection was set to be 40% for Omicron [9]. Note that when response lag is 3 weeks, cities with small population size may suffer from lockdown due to high proportion of population of cities were traced, and the outbreak is quickly contained, resulting in smaller number of tests.
(DOCX)

S10 Fig Effectiveness of population-level testing in China under 4-day testing interval (*R*_0_ = 10). Predicted epidemic duration and rounds of testing across 366 cities in China under different testing intervals and response lags when travel restrictions between cities is implemented with population-level testing. The grey dot represents the median and the grey error bar represents the 95% CI based on the 100 simulations. The dashed red line shows the duration of Wuhan’s lockdown in 2020 (i.e., 76 days) as reference. The number of cities with local outbreaks is labeled below the violin plot. The reduction of social distancing on transmission rate is set to 18% by considering the effect of only mask wearing against SARS-CoV-2 infection [8]. The vaccine coverage is set to 89% (consistent with 86% vaccine coverage in the ≥60 age group by August of 2022 in China) and the effectiveness of China’s inactivated vaccine (BBIBP-CorV and CoronaVac) against infection was set to be 40% for Omicron [9].
(DOCX)

S11 Fig Effectiveness of population-level testing combined with travel restrictions between cities in China during Omicron-like variant wave (*R*_0_ = 10). Predicted epidemic duration and rounds of testing across 366 cities in China under different testing intervals and response lags when travel restrictions between cities is implemented with population-level testing. The grey dot represents the median and the grey error bar represents the 95% CI based on the 100 simulations. The dashed red line shows the duration of Wuhan’s lockdown in 2020 (i.e., 76 days) as reference. The number of cities with local outbreaks is labeled below the violin plot. The reduction of social distancing on transmission rate is set to 18% by considering the effect of only mask wearing against SARS-CoV-2 infection [8]. The vaccine coverage is set to 89% (consistent with 86% vaccine coverage in the ≥60 age group by August of 2022 in China) and the effectiveness of China’s inactivated vaccine (BBIBP-CorV and CoronaVac) against infection was set to be 40% for Omicron [9].
(DOCX)

S12 Fig Effectiveness of population-level testing in China during Omicron-like variant wave when *R*_0_ = 8. Predicted epidemic duration and rounds of testing across 366 cities in China under different testing intervals and response lags when travel restrictions between cities is implemented with population-level testing. The grey dot represents the median and the grey error bar represents the 95% CI based on the 100 simulations. The dashed red line shows the duration of Wuhan’s lockdown in 2020 (i.e., 76 days) as reference. The number of cities with local outbreaks is labeled below the violin plot. The reduction of social distancing on transmission rate is set to 18% by considering the effect of only mask wearing against SARS-CoV-2 infection [8]. The vaccine coverage is set to 89% (consistent with 86% vaccine coverage in the ≥60 age group by August of 2022 in China) and the effectiveness of China’s inactivated vaccine (BBIBP-CorV and CoronaVac) against infection was set to be 40% for Omicron [9].
(DOCX)

S13 Fig Effectiveness of population-level testing in China during Omicron wave (*R*_0_ = 10) when rI = 2. Predicted epidemic duration and rounds of testing across 366 cities in China under different testing intervals and response lags when travel restrictions between cities is implemented with population-level testing. The grey dot represents the median and the grey error bar represents the 95% CI based on the 100 simulations. The dashed red line shows the duration of Wuhan’s lockdown in 2020 (i.e., 76 days) as reference. The number of cities with local outbreaks is labeled below the violin plot. The reduction of social distancing on transmission rate is set to 18% by considering the effect of only mask wearing against SARS-CoV-2 infection. The vaccine coverage is set to 89% (consistent with 86% vaccine coverage in the ≥60 age group by August of 2022 in China) and the effectiveness of China’s inactivated vaccine (BBIBP-CorV and CoronaVac) against infection was set to be 40% for Omicron.
(DOCX)

S14 Fig Comparison of epidemic duration and rounds of testing between two successive testing intervals without travel restrictions between cities (*R*_0_ = 10). Response lag is set to 2 weeks. A negative value corresponds to benefits yielded for duration or rounds by speeding up testing (shorten testing interval), and a positive value corresponds to potential loss for duration or rounds by speeding up testing. The red and blue dots represent median and grey error bar represents the 95% CI based on the 100 simulations. The reduction of social distancing on transmission rate is set to 18% by considering the effect of only mask wearing against SARS-CoV-2 infection [8]. The vaccine coverage is set to 89% (consistent with 86% vaccine coverage in the ≥60 age group by August of 2022 in China) and the effectiveness of China’s inactivated vaccine (BBIBP-CorV and CoronaVac) against infection was set to be 40% for Omicron [9].
(DOCX)

S15 Fig Comparison of epidemic duration and rounds of testing between two successive testing intervals when travel restrictions between cities is implemented (*R*_0_ = 10). **(A)** Response lag is set to 2 weeks. **(B)** Response lag is set to 3 weeks. A negative value corresponds to benefits yielded for duration or rounds by speeding up testing (shorten testing interval), and a positive value corresponds to potential loss for duration or rounds by speeding up testing. The red and blue dots represent median and grey error bar represents the 95% CI based on the 100 simulations. The reduction of social distancing on transmission rate is set to 18% by considering the effect of only mask wearing against SARS-CoV-2 infection [8]. The vaccine coverage is set to 89% (consistent with 86% vaccine coverage in the ≥60 age group by August of 2022 in China) and the effectiveness of China’s inactivated vaccine (BBIBP-CorV and CoronaVac) against infection was set to be 40% for Omicron [9].
(DOCX)

S16 Fig Potential impact of control strategy with long response lag on SARS-CoV-2 infections and daily life. (**A**) The total SARS-CoV-2 infections for different age groups when testing interval is 3 days and response lag is 3 weeks. The dot represents the infections for an age group and a city. (**B**) Association between population size and proportion of isolated population across 366 cities in China when testing interval is 3 days and response lag is 3 weeks. The size and color of the circle represent the movement flow and the proportion of isolated population in a given city, respectively.
(DOCX)

S17 Fig COVID-19 burden for Omicron-like variant (*R*_0_ = 10) under 4-day testing interval. (**A**) Daily required hospital beds for different age groups. (**B**) Daily required ICU beds for different age groups. The response lag is set to be 3 weeks. The grey error bar or shadow represents the 95% CI for 100 simulations. The red dashed line represents the total available hospital beds or ICU beds in China. The vaccine coverage for all age groups was set to be 89%, consistent with 86% vaccine coverage in the ≥60 age group by August of 2022 in China.
(DOCX)

S18 Fig COVID-19 burden for Omicron-like variant (*R*_0_ = 10) under the control strategy in China for optimistic scenario. (**A**) Daily required hospital beds for different age groups. (**B**) Daily required ICU beds for different age groups. The control strategy was employed with a testing interval of 3 days and a response lag of 3 weeks (the effective reproduction number *R*_e_ < 1), which is the least stringent strategy aiming at suppressing SARS-CoV-2, as shown in Fig 2A. A strategy with a testing interval of 4 days and a response lag of 3 weeks would lead to the endemic of COVID-19 as shown in the grey dashed line with *R*_e_ ≈ 1. The red dashed line represents the total available hospital beds or ICU beds in China. The grey solid line represents the peak number of required hospital beds or ICU beds during the pandemic without testing (*R*_e_ > 1). The grey error bar or shadow represents the 95% CI for 100 simulations. The vaccine coverage for all age groups was set to be 89%, consistent with 86% vaccine coverage in the ≥60 age group by August of 2022 in China. The effectiveness of China’s inactivated vaccine (BBIBP-CorV and CoronaVac) against hospitalization and ICU admission were set to be 78.8% considering an optimistic scenario [10,11].
(DOCX)

S19 Fig COVID-19 burden for Omicron-like variant (*R*_0_ = 10) under the control strategy in China for pessimistic scenario. (**A**) Daily required hospital beds for different age groups. (**B**) Daily required ICU beds for different age groups. The control strategy was employed with a testing interval of 3 days and a response lag of 3 weeks (the effective reproduction number *R*_e_ < 1), which is the least stringent strategy aiming at suppressing SARS-CoV-2, as shown in Fig 2A. A strategy with a testing interval of 4 days and a response lag of 3 weeks would lead to the endemic of COVID-19 as shown in the grey dashed line with *R*_e_ ≈ 1. The red dashed line represents the total available hospital beds or ICU beds in China. The grey solid line represents the peak number of required hospital beds or ICU beds during the pandemic without testing (*R*_e_ > 1). The grey error bar or shadow represents the 95% CI for 100 simulations. The vaccine coverage for all age groups was set to be 89%, consistent with 86% vaccine coverage in the ≥60 age group by August of 2022 in China. The effectiveness of China’s inactivated vaccine (BBIBP-CorV and CoronaVac) against hospitalization and ICU admission were set to be 62.6% considering an pessimistic scenario [10,11].
(DOCX)

S20 Fig COVID-19 burden for Omicron-like variant (*R*_0_ = 10) under the control strategy in China for Hong Kong-specific age-dependent hospitalization and ICU admission rates. **(A)** Daily required hospital beds for different age groups. **(B)** Daily required ICU beds for different age groups. The control strategy was employed with a testing interval of 3 days and a response lag of 3 weeks (the effective reproduction number *R*_e_ < 1), which is the least stringent strategy aiming at suppressing SARS-CoV-2, as shown in Fig 2A. A strategy with a testing interval of 4 days and a response lag of 3 weeks would lead to the endemic of COVID-19 as shown in the grey dashed line with *R*_e_ ≈ 1. The red dashed line represents the total available hospital beds or ICU beds in China. The grey dotted line represents the strategy with a testing interval of 4 days and a response lag of 3 weeks. The grey solid line represents the peak number of required hospital beds or ICU beds during the pandemic without testing (*R*_e_ > 1). The grey error bar or shadow represents the 95% CI for 100 simulations. The vaccine coverage for all age groups was set to be 89%, consistent with 86% vaccine coverage in the ≥60 age group by August of 2022 in China. The age-dependent hospitalization and ICU admission rates were set according to data from the fifth wave of COVID-19 in Hong Kong in early 2022 [12].
(DOCX)

S21 Fig COVID-19 burden for Omicron-like variant (*R*_0_ = 10) under the control strategy in China for Shanghai-specific age-dependent ICU admission rates. Daily required ICU beds for different age groups. The control strategy was employed with a testing interval of 3 days and a response lag of 3 weeks (the effective reproduction number *R*_e_ < 1), which is the least stringent strategy aiming at suppressing SARS-CoV-2, as shown in Fig 2A. A strategy with a testing interval of 4 days and a response lag of 3 weeks would lead to the endemic of COVID-19 as shown in the grey dashed line with *R*_e_ ≈ 1. The red dashed line represents the total available ICU beds in China. The grey dotted line represents the strategy with a testing interval of 4 days and a response lag of 3 weeks. The grey solid line represents the peak number of required ICU beds during the pandemic without testing (*R*_e_ > 1). The grey error bar or shadow represents the 95% CI for 100 simulations. The vaccine coverage for all age groups was set to be 89%, consistent with 86% vaccine coverage in the ≥60 age group by August of 2022 in China. The age-dependent ICU admission rates were set according to data from the Omicron wave of COVID-19 in Shanghai in early 2022 [13]. Due to the lack of hospitalization data in Omicron wave of COVID-19 in Shanghai, only the ICU burden of COVID-19 for the Omicron variant was evaluated under this scenario.
(DOCX)

S22 Fig Potential impact of control strategy with short response lag on SARS-CoV-2 infections and daily life. (**A**) The total SARS-CoV-2 infections for different age groups when testing interval is 3 days and response lag is 2 weeks. The dot represents the infections for an age group and a city. (**B**) Association between population size and proportion of isolated population across 366 cities in China when testing interval is 3 days and response lag is 2 weeks. The size and color of the circle represent the movement flow and the proportion of isolated population in a given city, respectively.
(DOCX)

S23 Fig The disease burden for Omicron-like variant (*R*_0_ = 10) outbreak without considering the underlying health conditions. (**A**) The number of hospitalizations and (**B**) ICU admissions when the least stringent control strategy is employed. We considered all the infected individuals were healthy in the baseline scenario. The grey error bar or shadow represents the 95% CI for 100 simulations.
(DOCX)

S1 Table The outbreaks controlled by public health measures in China. (DOCX)

S2 Table Estimated parameters values in the first wave of 2020 in China. (DOCX)

S3 Table The significance of reduction in epidemic duration and rounds of testing by shorter response lag (weeks) from the *t*-test. (DOCX)

S4 Table The significance of reduction in epidemic duration and rounds of testing by shorter testing interval from the *t*-test. (DOCX)

S5 Table The number of cities with the peak number of isolated individuals reaching the population size during the outbreak under different combinations of testing intervals and response lags. The isolated individuals include the individuals exposed to the SARS-CoV-2 and the infected individuals.
(DOCX)

S6 Table Baseline model parameter values. (DOCX)

S7 Table Population-level testing and contact tracing model parameter values. (DOCX)

S8 Table Age-dependent hospitalization and ICU admission rates for symptomatic Omicron- infection in vaccinated and unvaccinated individuals. The effectiveness of China’s inactivated vaccine (BBIBP-CorV and CoronaVac) against hospitalization and ICU admission were set to be 70% for Omicron for all age groups [11].
(DOCX)
