# Supplementary material for: Marginal effects of public health measures and COVID-19 disease burden in China: A large-scale modelling study
Source: PLoS Comput Biol. 2023 Sep 18;19(9):e1011492. doi: 10.1371/journal.pcbi.1011492 (PMC10538769; doi:10.1371/journal.pcbi.1011492)
Supplement: S1 Text — (DOCX) [file pcbi.1011492.s001.docx]

**Supplementary Materials for**

**Marginal effects of public health measures and COVID-19 disease burden in China: a large-scale modelling study**

**Authors:** Zengmiao Wang^1^†*, Peiyi Wu^1^†, Lin Wang^2^, Bingying Li^1^, Yonghong Liu^3^, Yuxi Ge^1^, Ruixue Wang^1^, Ligui Wang^4^, Hua Tan^5^, Chieh-Hsi Wu^6^, Marko Laine^7^, Henrik Salje^2^, Hongbin Song^4^*

**Affiliations:**

^1^ State Key Laboratory of Remote Sensing Science, Center for Global Change and Public Health, Faculty of Geographical Science, Beijing Normal University, Beijing, China

^2^ Department of Genetics, University of Cambridge, Cambridge, UK

^3^ Beijing Center for Disease Prevention and Control, Beijing, China

^4^ Center of Disease Control and Prevention, PLA, Beijing, China

^5^ Translational and Functional Genomics Branch, National Human Genome Research Institute, National Institutes of Health, Bethesda, Maryland, United States of America

^6^ Mathematical Sciences, University of Southampton, Southampton, United Kingdom

^7^ Finnish Meteorological Institute, Meteorological Research Unit, Helsinki, Finland

* Correspondence and requests for materials should be addressed to Z.W. (wangzengmiao@gmail.com) or H.S. (hongbinsong@263.net)

† These authors contributed equally to this work.

**This PDF file includes:**

Supplementary text

Reference

**Overview for the Supplementary Materials**

The texts of Supplementary Materials are organized as follows. In Section S1, three metapopulation models were developed with increasing complexity. First, the baseline metapopulation model integrated inter-city movements across 366 Chinese cities was introduced to set up the context of modelling in Section S1.1. In this model, SARS-CoV-2 spread naturally without any NPIs and COVID-19 vaccines. Second, we extended the baseline model by considering the social distancing, travel restriction between cities and vaccination in Section S1.2. The extended model was fitted using the real data from the first SARS-CoV-2 wave of 2020 in China. During the fitting, the vaccination was set to zero because no COVID-19 vaccines were available at that time. Third, in Section S1.3, we developed a stochastic age-stratified metapopulation model that included population-level testing, contact tracing, vaccination and human mobility across 366 cities to illustrate the effectiveness of public health measures in a highly transmissible variant wave. In Section S2, the effectiveness of the initial version of public health measures against Wuhan-Hu-1 and a highly transmissible variant like Omicron were assessed by varying the strength of social distancing and travel restrictions based on the fitted model of Section S1.2. In Section S3, the effects of the 8 types of underlying health conditions on the hospitalizations and ICU admissions were evaluated based on the age-stratified metapopulation model. In Section S4, the calculation procedures for the parameters used in this study were introduced.

**S1. Metapopulation models**

**S1.1.** **Baseline metapopulation model**

We developed a metapopulation model integrated inter-city movements across 366 Chinese cities to reconstruct the transmission dynamic of SARS-CoV-2. In the baseline scenario, SARS-CoV-2 spread naturally without any NPIs and vaccination. The full equations of baseline model are given by:

$$S_{i}\left( t+1 \right)=S_{i}\left( t \right)-\lambda_{i}^{S}\left( t \right)\frac{S_{i}\left( t \right)}{N_{i}}+\sum_{j} T_{ji}^{S}\left( t \right)-\sum_{j} T_{ij}^{S}\left( t \right)$$

$$E_{i}\left( t+1 \right)=E_{i}\left( t \right)+\lambda_{i}^{S}\left( t \right)\frac{S_{i}\left( t \right)}{N_{i}}-\gamma^{E}E_{i}(t)+\sum_{j} T_{ji}^{E}(t)-\sum_{j} T_{ij}^{E}(t)$$

$$A_{i}(t+1)=A_{i}(t)+p_{a}\gamma^{E}E_{i}(t)-\gamma^{A}A_{i}(t)+\sum_{j} T_{ji}^{A}(t)-\sum_{j} T_{ij}^{A}(t)$$

$$P_{i}(t+1)=P_{i}(t)+(1-p_{a})\gamma^{E}E_{i}(t)-\gamma^{P}P_{i}(t)+\sum_{j} T_{ji}^{P}(t)-\sum_{j} T_{ij}^{P}(t)$$

$$I_{i}(t+1)=I_{i}(t)+\gamma^{P}P_{i}(t)-\gamma^{I}I_{i}(t)$$

$$R_{i}\left( t+1 \right)=R_{i}\left( t \right)+\gamma^{A}A_{i}\left( t \right)+\gamma^{I}I_{i}\left( t \right)$$

$$N_{i}\left( t \right)=S_{i}\left( t \right)+E_{i}(t)+P_{i}(t)+A_{i}(t)+I_{i}(t)+R_{i}(t)$$

where

$$\lambda_{i}^{S}\left( t \right)=\lambda_{{basic}_{i}}\left( t \right)=\beta^{A}A_{i}\left( t \right)+\beta^{P}P_{i}\left( t \right)+\beta^{I}I_{i}\left( t \right)$$

$$T_{ji}^{X}\left( t \right)=T_{{basic}_{ji}}^{X}\left( t \right)=\frac{X_{j}\left( t \right)}{N_{j}}T_{ji}\left( t \right), X\in\{S,E,A,P\}$$

Different states are considered as susceptible (*S_i_*), latent (*E_i_*), pre-symptomatic (*P_i_*), infectious asymptomatic (*A_i_*), infectious symptomatic (*I_i_*), and recovered (*R_i_*) individuals. The subscripts *i* and *j* refer to the city ID, and *t* refers to time in day. $p_{a}$ is the proportion of asymptomatic cases. 1/$\gamma^{E}$ is the latent period. 1/$\gamma^{P}$is the pre-symptomatic period. 1/$\gamma^{A}$ represents the infectious period for asymptomatic individuals. 1/$\gamma^{I}$ represents the infectious period for symptomatic individuals. Basic force of infection $\lambda_{{basic}_{i}}$ of SARS-CoV-2 depends on the number of infectious individuals in *P, A, I* and their transmission rates. Let $\beta^{I}$ represent the transmission rate for the infectious symptomatic individuals. We take the transmission rate $\beta^{A}$ for infectious asymptomatic individuals to be $r_{1}\beta^{I}$ and the transmission rate $\beta^{P}$ for pre-symptomatic individuals to be $r_{2}\beta^{I}$ , similar to the setting of [1]. The value of each transmission dynamic parameter is set according to S6 Table. According to the next generation matrix, $\beta_{I}=\frac{R_{0}}{p_{a}\frac{r_{1}}{\gamma^{A}}+\left( 1-p_{a} \right)\left( \frac{r_{2}}{\gamma^{P}}+\frac{1}{\gamma^{I}} \right)}$.

$T_{ji}(t)$ is the number of people leaving city *j* on day *t* travelling to city *i*. Different states (excluding infectious symptomatic and recovered individuals) in travel movements are also considered. $T_{{basic}_{ji}}^{X}\left( t \right)$ represents the number of individuals in compartment $X\in\{S,E,A,P\}$ imported from city *j* to city *i* on day *t* under baseline travel movements.

**S1.2. Metapopulation model with the** **social distancing, travel restriction between cities and vaccination**

Advanced model with the social distancing, travel restriction between cities and vaccination is built based on the baseline model, considering vaccinated individuals (*V_i_*) and detected and isolated individuals (*D_i_*). Due to testing methods and testing capabilities, infected individuals are detected only in symptomatic individuals. If symptomatic individuals tested positive, they would be isolated, treated and exit the transmission chain. The full equations of advanced model are given by:

$$S_{i}\left( t+1 \right)=S_{i}\left( t \right)-\lambda_{i}^{S}\left( t \right)\frac{S_{i}\left( t \right)}{N_{i}}+\sum_{j} T_{ji}^{S}\left( t \right)-\sum_{j} T_{ij}^{S}\left( t \right)$$

$$V_{i}\left( t+1 \right)=V_{i}\left( t \right)-\lambda_{i}^{V}\left( t \right)\frac{V_{i}\left( t \right)}{N_{i}}+\sum_{j} T_{ji}^{V}\left( t \right)-\sum_{j} T_{ij}^{V}\left( t \right)$$

$$E_{i}\left( t+1 \right)=E_{i}\left( t \right)+\lambda_{i}^{S}\left( t \right)\frac{S_{i}\left( t \right)}{N_{i}}+\lambda_{i}^{V}\left( t \right)\frac{V_{i}\left( t \right)}{N_{i}}-\gamma^{E}E_{i}(t)+\sum_{j} T_{ji}^{E}(t)-\sum_{j} T_{ij}^{E}(t)$$

$$A_{i}(t+1)=A_{i}(t)+p_{a}\gamma^{E}E_{i}(t)-\gamma^{A}A_{i}(t)+\sum_{j} T_{ji}^{A}(t)-\sum_{j} T_{ij}^{A}(t)$$

$$P_{i}(t+1)=P_{i}(t)+(1-p_{a})\gamma^{E}E_{i}(t)-\gamma^{P}P_{i}(t)+\sum_{j} T_{ji}^{P}(t)-\sum_{j} T_{ij}^{P}(t)$$

$$I_{i}(t+1)=I_{i}(t)+\gamma^{P}P_{i}(t)-\left( \gamma^{I}+\delta\right)I_{i}(t)$$

$$R_{i}\left( t+1 \right)=R_{i}\left( t \right)+\gamma^{A}A_{i}\left( t \right)+\gamma^{I}I_{i}\left( t \right)$$

$$D_{i}\left( t \right)=\delta I_{i}\left( t \right)$$

$$N_{i}\left( t \right)=S_{i}\left( t \right)+V_{i}\left( t \right)+E_{i}(t)+P_{i}(t)+A_{i}\left( t \right)+I_{i}\left( t \right)+R_{i}\left( t \right)+D_{i}\left( t \right)$$

where

$$\lambda_{i}^{S}\left( t \right)=(1-\beta_{C}){\times\lambda}_{{basic}_{i}}\left( t \right)$$

$$\lambda_{i}^{V}\left( t \right)=\left( 1-\varepsilon\right)\times\left( 1-\beta_{C} \right)\times\lambda_{{basic}_{i}}\left( t \right)$$

$$T_{ji}^{X}\left( t \right)=B_{ji}\left( t \right)T_{{basic}_{ji}}^{X}\left( t \right), X\in\{S,E,A,P,V\}$$

*δ* is the detecting rate of symptomatic individuals. Following the implementation of national emergency response in China on January 25th, 2020, the testing capacity was significantly increased. Consequently, we fitted different detection rates before and after this date. To account for the varying degrees of COVID-19 severity across China, we tailored the detection rates for Wuhan city, other cities in Hubei province, and cities in China (excluding Hubei), using different rates for each category. Additionally, we assumed a constant ratio between the detection rates of other cities in Hubei province and cities in China (excluding Hubei) after national emergency response. Before national emergence response, this ratio was set to 1. *β_C_* is the effect of social distancing on the reduction of transmission rate. $\varepsilon$ is the vaccine effectiveness against infection. $B_{ji}\left( t \right)$ is the effect of travel restriction on travel movements of individuals in compartment $X\in\{S,E,A,P, V\}$ between city *j* and city *i* on day *t*. Other compartments and parameters were consistent with baseline model, please refer to S6 Table.

To reconstruct the spatial spread of SARS-CoV-2 in the first wave of 2020 in China, we fitted $D_{i}\left( t \right)$ to the daily new cases in each city of China from January 1 to March 6, 2020. Real-time inter-city travel data from January to March 2020 were used. The social distancing (*β_C_*) is considered to be carried out simultaneously within each province and starts when more than 95% of cities in a province had implemented control measures. Model fitting was performed using the Metropolis–Hastings Markov chain Monte Carlo (MCMC) algorithm with the MATLAB (version R2020a) toolbox mcmcstat [2] (https://github.com/mjlaine/mcmcstat). During the fitting, the vaccinated individuals (*V_i_*) was set to zero because no COVID-19 vaccines were available at that time. The dynamics parameters of SARS-CoV-2 were set based on S6 Table.

After a burn-in of 2 million iterations, we ran the MCMC simulation for a further 5 million iterations, sampled at every 1000th step to avoid auto-correlation. Trace plots and Gelman and Rubin diagnostics were used to judge convergence of the MCMC chains. Each fitting exercise was repeated three times to test the robustness of results, which converged to the same estimates all occasions. The estimated parameters are shown in the S2 Table. The consistency between the predicted daily COVID-19 cases and observed daily cases during the first wave demonstrates the capability of the developed model (Figs S5–S6).

**S1.3. Age-stratified metapopulation model with population-level testing and contact tracing**

In this section, we extended above model and introduced a stochastic age-stratified population-level testing and contract tracing model. Specifically, we considered susceptible (*S_i,m_*), latent (*E_i,m_*), pre-symptomatic (*P_i,m_*), infectious asymptomatic (*A_i,m_*), infectious symptomatic (*I_i,m_*), recovered (*R_i,m_*) individuals, hospitalized patients ($H_{i,m}^{baseline}$), ICU patients ($U_{i,m}^{baseline}$). The subscript *i* refers to the city ID, consistent with previous models, and subscript *m* refers to the age group. The population is divided into 4 age groups: 0-19, 20-39, 40-59, ≥60. Three compartments related to population-level testing and contact tracing were added: infected individuals detected through population-level testing (*T_i,m_*), infected individuals detected through contact tracing (*C_i,m_*), and healthy susceptible individuals traced and isolated ($Q_{i,m}^{S}$). Vaccination is also considered, represented by vaccinated individuals (*V_i,m_*) and healthy vaccinated individuals traced and isolated ($Q_{i,m}^{V}$). If individuals tested positive, they would be isolated and exit the transmission chain. Healthy individuals who have close contact with infected individuals may also be traced and isolated. The full equations of population-level testing and contact tracing model are given by:

$$S_{i,m}\left( t+1 \right)=S_{i,m}\left( t \right)-W_{i,m}^{S}\left( t \right)-\mu_{i,m}(t)\frac{S_{i,m}\left( t \right)}{N_{i,m}}+qQ_{i,m}^{S}\left( t \right)+\sum_{j} T_{ji,m}^{S}\left( t \right)-\sum_{j} T_{ij,m}^{S}\left( t \right)$$

$$V_{i,m}\left( t+1 \right)=V_{i,m}\left( t \right)-W_{i,m}^{V}(t)-\mu_{i,m}(t)\frac{V_{i,m}\left( t \right)}{N_{i,m}}+qQ_{i,m}^{V}\left( t \right)+\sum_{j} T_{ji,m}^{V}\left( t \right)-\sum_{j} T_{ij,m}^{V}\left( t \right)$$

$$E_{i,m}\left( t+1 \right)=E_{i,m}\left( t \right)+W_{i,m}^{S}\left( t \right)+W_{i,m}^{V}(t)-\left( {\pi^{E}\tau}_{i}\left( t \right)+\left( 1-{\pi^{E}\tau}_{i}\left( t \right) \right)\gamma^{E} \right)E_{i,m}\left( t \right)-\pi^{I}\tau_{i}\left( t \right)\kappa L\left\{ \sum_{n=1}^{G} M_{mn}I_{i,n}\theta_{i,mn}^{I}p_{i,n}^{IE} \right\}-\pi^{P}\tau_{i}\left( t \right)\kappa L\left\{ \sum_{n=1}^{G} M_{mn}P_{i,n}\theta_{i,mn}^{P}p_{i,n}^{PE} \right\}-\pi^{A}\tau_{i}(t)\kappa L\{\sum_{n=1}^{G} M_{mn}A_{i,n}\theta_{i,mn}^{A}p_{i,n}^{AE}\}-\mu_{i,m}(t)\frac{E_{i,m}\left( t \right)}{N_{i,m}}+\sum_{j} T_{ji,m}^{E}(t)-\sum_{j} T_{ij,m}^{E}(t)$$

$$A_{i,m}\left( t+1 \right)=A_{i,m}\left( t \right)+p_{a.m}\left( 1-{\pi^{E}\tau}_{i}\left( t \right) \right)\gamma^{E}E_{i,m}\left( t \right)-\left( \pi^{A}\tau_{i}\left( t \right)+\left( 1-{\pi^{A}\tau}_{i}\left( t \right) \right)\gamma^{A} \right)A_{i,m}\left( t \right)-\pi^{I}\tau_{i}\left( t \right)\kappa L\left\{ \sum_{n=1}^{G} M_{mn}I_{i,n}\theta_{i,mn}^{I}p_{i,n}^{IA} \right\}-\pi^{P}\tau_{i}\left( t \right)\kappa L\left\{ \sum_{n=1}^{G} M_{mn}P_{i,n}\theta_{i,mn}^{P}p_{i,n}^{PA} \right\}-\pi^{A}\tau_{i}(t)\kappa L\{\sum_{n=1}^{G} M_{mn}A_{i,n}\theta_{i,mn}^{A}p_{i,n}^{AA}\}-\mu_{i,m}(t)\frac{A_{i,m}\left( t \right)}{N_{i,m}}+\sum_{j} T_{ji,m}^{A}(t)-\sum_{j} T_{ij,m}^{A}(t)$$

$$P_{i,m}\left( t+1 \right)=P_{i,m}\left( t \right)+\left( 1-p_{a,m} \right)\left( 1-{\pi^{E}\tau}_{i}\left( t \right) \right)\gamma^{E}E_{i,m}\left( t \right)-\left( \pi^{P}\tau_{i}\left( t \right)+\left( 1-{\pi^{P}\tau}_{i}\left( t \right) \right)\gamma^{P} \right)P_{i,m}\left( t \right)-\pi^{I}\tau_{i}\left( t \right)\kappa L\left\{ \sum_{n=1}^{G} M_{mn}I_{i,n}\theta_{i,mn}^{I}p_{i,n}^{IP} \right\}-\pi^{P}\tau_{i}\left( t \right)\kappa L\left\{ \sum_{n=1}^{G} M_{mn}P_{i,n}\theta_{i,mn}^{P}p_{i,n}^{PP} \right\}-\pi^{A}\tau_{i}(t)\kappa L\{\sum_{n=1}^{G} M_{mn}A_{i,n}\theta_{i,mn}^{A}p_{i,n}^{AP}\}-\mu_{i,m}(t)\frac{P_{i,m}\left( t \right)}{N_{i,m}}+\sum_{j} T_{ji,m}^{P}(t)-\sum_{j} T_{ij,m}^{P}(t)$$

$$I_{i,m}\left( t+1 \right)=I_{i,m}\left( t \right)+\left( 1-{\pi^{P}\tau}_{i}\left( t \right) \right)\gamma_{P}P_{i,m}\left( t \right)-\left( \pi^{I}\tau_{i}\left( t \right)+\left( 1-{\pi^{I}\tau}_{i}\left( t \right) \right)\gamma^{I} \right)I_{i,m}\left( t \right)-\pi^{I}\tau_{i}\left( t \right)\kappa L\left\{ \sum_{n=1}^{G} M_{mn}I_{i,n}\theta_{i,mn}^{I}p_{i,n}^{II} \right\}-\pi^{P}\tau_{i}\left( t \right)\kappa L\left\{ \sum_{n=1}^{G} M_{mn}P_{i,n}\theta_{i,mn}^{P}p_{i,n}^{PI} \right\}-\pi^{A}\tau_{i}\left( t \right)\kappa L\left\{ \sum_{n=1}^{G} M_{mn}A_{i,n}\theta_{i,mn}^{A}p_{i,n}^{AI} \right\}-\mu_{i,m}\left( t \right)\frac{I_{i,m}\left( t \right)}{N_{i,m}}$$

$$R_{i,m}\left( t+1 \right)=R_{i,m}\left( t \right)+\left( 1-{\pi^{A}\tau}_{i}(t) \right)\gamma^{A}A_{i,m}\left( t \right)+\left( 1-{\pi^{I}\tau}_{i}(t) \right)\gamma^{I}I_{i,m}\left( t \right)$$

$$Q_{i,m}^{S}\left( t+1 \right)=Q_{i,m}^{S}\left( t \right)-qQ_{i,m}^{S}\left( t \right)+\mu_{i,m}(t)\frac{S_{i,m}\left( t \right)}{N_{i,m}}$$

$$Q_{i,m}^{V}\left( t+1 \right)=Q_{i,m}^{V}\left( t \right)-qQ_{i,m}^{V}\left( t \right)+\mu_{i,m}(t)\frac{V_{i,m}\left( t \right)}{N_{i,m}}$$

$$C_{i,m}\left( t+1 \right)=C_{i,m}\left( t \right)+\pi^{I}\tau_{i}(t)\kappa L\{\sum_{n=1}^{G} M_{mn}I_{i,n}\theta_{i,mn}^{I}\}+\pi^{P}\tau_{i}(t)\kappa L\{\sum_{n=1}^{G} M_{mn}P_{i,n}\theta_{i,mn}^{P}\}+\pi^{A}\tau_{i}(t)\kappa L\{\sum_{n=1}^{G} M_{mn}A_{i,n}\theta_{i,mn}^{A}\}+\mu_{i,m}(t)\frac{E_{i,m}\left( t \right)+A_{i,m}\left( t \right)+P_{i}\left( t \right)+I_{i,m}\left( t \right)}{N_{i,m}}$$

$$T_{i,m}\left( t+1 \right)=T_{i,m}\left( t \right)+\left( \pi^{E}E_{i,m}\left( t \right)+\pi^{A}A_{i,m}\left( t \right)+\pi^{P}P_{i,m}\left( t \right)+\pi^{I}I_{i,m}\left( t \right) \right)\tau_{i,m}(t)$$

$$N_{i,m}(t)=S_{i,m}\left( t \right)+V_{i,m}\left( t \right)+E_{i,m}(t)+P_{i,m}(t)+A_{i,m}\left( t \right)+I_{i,m}\left( t \right)+R_{i,m}\left( t \right)+Q_{i,m}^{S}\left( t \right)+Q_{i,m}^{V}\left( t \right)+C_{i,m}\left( t \right)+T_{i,m}\left( t \right)$$

$$H_{i,m}^{baseline}\left( t+1 \right)=H_{i,m}^{baseline}\left( t \right)+P_{m}^{S\to H}\sum_{tlSH=0}^{lSH-1} \frac{1}{lSH}W_{i,m}^{S}\left( t-tlSH \right)+P_{m}^{V\to H}\sum_{tlVH=0}^{lVH-1} {\frac{1}{lVH}W}_{i,m}^{V}\left( t-tlVH \right)-P_{m}^{S\to H}\sum_{tlSH=0}^{lSH-1} \frac{1}{lSH}W_{i,m}^{S}\left( t-tlSH-lSHR \right)-P_{m}^{V\to H}\sum_{tlVH=0}^{lVH-1} {\frac{1}{lVH}W}_{i,m}^{V}\left( t-tlVH-lVHR \right)$$

$$U_{i,m}^{baseline}\left( t+1 \right)=U_{i,m}^{baseline}\left( t \right)+P_{m}^{S\to U}\sum_{tlSU=0}^{lSU-1} \frac{1}{lSU}W_{i,m}^{S}\left( t-tlSU \right)+P_{m}^{V\to U}\sum_{tlVU=0}^{lVU-1} \frac{1}{lVU}W_{i,m}^{V}\left( t-tlVU \right)-P_{m}^{S\to U}\sum_{tlSU=0}^{lSU-1} \frac{1}{lSU}W_{i,m}^{S}\left( t-tlSU-lSUR \right)-P_{m}^{V\to U}\sum_{tlVU=0}^{lVU-1} \frac{1}{lVU}W_{i,m}^{V}\left( t-tlVU-lVUR \right)$$

where

$$\lambda_{{basic}_{i,m}}\left( t \right)=\frac{1}{N_{m}}\sum_{n=1}^{G} M_{mn}\left( \beta^{A}A_{i,n}\left( t \right)+\beta^{P}P_{i,n}\left( t \right)+\beta^{I}I_{i,n}\left( t \right) \right)$$

$$\lambda_{i,m}^{S}\left( t \right)=(1-\beta_{C})\lambda_{{basic}_{i,m}}\left( t \right)$$

$\lambda_{i,m}^{V}\left( t \right)=\left( 1-\varepsilon\right)\left( 1-\beta_{C} \right)\lambda_{{basic}_{i,m}}\left( t \right)$

$$W_{i,m}^{S}\left( t \right)\sim B(S_{i,m}\left( t \right),p=1-exp(-\lambda_{i,m}^{S}\left( t \right)))$$

$$W_{i,m}^{V}\left( t \right)\sim B(V_{i,m}\left( t \right),p=1-exp(-\lambda_{i,m}^{V}\left( t \right)))$$

$$T_{{basic}_{ji,m}}^{X}\left( t \right)=\frac{X_{j,m}\left( t \right)}{N_{j,m}}T_{ji,m}\left( t \right), X\in\left\{ S,E,A,P,V \right\}$$

$$T_{ji,m}^{X}\left( t \right)=B_{ji}(t)T_{{basic}_{ji,m}}^{X}\left( t \right), X\in\{S,E,A,P,V\}$$

$\theta_{i,mn}^{X}\left( t \right)=\frac{(1-\beta_{C})\hat{\beta^{X}}M_{mn}S_{i,m}(t)+(1-\beta_{C})\left( 1-\varepsilon\right)\hat{\beta^{X}}M_{mn}V_{i,m}(t)}{N_{m}M_{mn}}, X\in\{A,P,I\}$

$${\mu.}_{i,m}=\pi^{I}\tau_{i}(t)\kappa L\{\sum_{n=1}^{G} M_{mn}I_{n}(1-\theta_{i,mn}^{I})\}+\pi^{P}\tau_{i}(t)\kappa L\{\sum_{n=1}^{G} M_{mn}P_{n}{(1-\theta}_{i,mn}^{P})\}+\pi^{A}\tau_{i}(t)\kappa L\{\sum_{n=1}^{G} M_{mn}A_{n}(1-\theta_{i,mn}^{A})\}+\pi^{E}\tau_{i}(t)\kappa L\{\sum_{=1}^{G} M_{mn}E_{n}\}$$

$$P_{m}^{S\to H}=p_{h,m}^{unvac}\left( 1-p_{a,m} \right)$$

$$P_{m}^{V\to H}=p_{h,m}^{vac}\left( 1-p_{a,m} \right)$$

$$P_{m}^{S\to U}=p_{h,m}^{unvac}p_{u,m}^{unvac}\left( 1-p_{a,m} \right)$$

$$P_{m}^{V\to U}=p_{h,m}^{vac}p_{u,m}^{vac}\left( 1-p_{a,m} \right)$$

$$lSH=lVH=\frac{1}{\left( \gamma^{E}+\gamma^{P}+\gamma^{I2H} \right)}$$

$$lSHR=lVHR=\frac{1}{\gamma^{H}}$$

$$lSU=lVU=\frac{1}{\left( \gamma^{E}+\gamma^{P}+\gamma^{I2U} \right)}$$

$$lSUR=lVUR=\frac{1}{\gamma^{U}}$$

*B* (*n*, *p*) denotes a binomial distribution with *n* trials each with probability of success *p*. The chain-binomial was used in this stochastic model [3]. 1/$\gamma^{I2H} and 1/\gamma^{I2U}$ is the average time from symptom onset to hospitalization or ICU admission. 1/$\gamma^{H} and 1/\gamma^{U}$ is length of hospital or ICU stay before recovery. This model focus on the population-level testing. Therefore, the number of hospitalized patients $H_{i,m}^{baseline}\left( t \right)$ and the number of people admitted to ICU $U_{i,m}^{baseline}\left( t \right)$ are not easily measured. We considered them calculated from the daily new infections. We assume that a fraction $P_{m}^{S\to H}$ of $W_{i,m}^{S}\left( t \right)$ will be admitted into hospital after a delay (ranged from 0 to $lSH-1$) from the exposure. And, a fraction $P_{m}^{S\to H}$ of $W_{i,m}^{S}\left( t \right)$ will be discharged after a delay (ranged from $lSHR$ to $lSH-1+lSHR$) from the exposure. $H_{i,m}^{baseline}\left( t \right)$ is the difference between the hospitalized patients and the discharged patients. Similarly, $U_{i,m}^{baseline}\left( t \right)$ is also obtained. $p_{h,m}^{vac}$ and $p_{h,m}^{unvac}$ are the proportion of symptomatic infections requiring hospitalizations in age group *m* for vaccinated and unvaccinated individuals, and $p_{u,m}^{vac}$ and $p_{u,m}^{unvac}$ are the proportion of hospitalized patients requiring ICU in age group *m* for vaccinated and unvaccinated individuals, please refer to S8 Table. 1/*q* is duration of isolation. Considering that the sensitivity of PCR test depends on the disease progress, the PCR test sensitivities for different compartments were included. Specifically, $\pi^{E},\pi^{A},\pi^{P},\pi^{I}$represents the sensitivity of PCR tests for the individuals in compartment *E*, *A*, *P* and *I*, respectively. As implementation of other basic NPIs (for example, wearing face masks) and deployment of vaccines, the force of infection was calculated under social distancing *β_C_* and vaccine effectiveness *ε*. In our simulation, we assumed that China’s inactivated vaccine (BBIBP-CorV and CoronaVac) are distributed. Travel restriction $B_{ji}\left( t \right)$ on movements between city *j* and city *i* on day *t* is also considered.

We modeled the CT like those in the study of Davin Lunz et al [4] with extension. The contact tracing rate for the compartments on day *t* in city *i* in age group *m* is given by the testing rate represented by $\tau_{i}(t)$, the sensitivity of PCR test, the fraction of contacts that were successfully traced represented by $\kappa$, the contact number of age group *m* with other age group *n* represented by $M_{mn}$, and the pre-defined CT time window (L days). Therefore, the contact tracing rate is $\pi^{X}\tau_{i}\left( t \right)\kappa L\left\{ \sum_{n=1}^{G} M_{mn}X_{i,n} \right\}, X\in\{A,P,I\}$. $\kappa$ depends on the strictness and capacity of CT in a city. *L* depends on the specific infectious disease.

Next, the traced contacts in city *i* in age group *m* who were truly infected by the primary cases from compartment $X\in\{A,P,I\}$ on day *t* were modeled*.* Let $\theta_{i,mn}^{X}(t)$ be the contact tracing precision in city *i* in age group *m* for the primary cases in age group *n* from compartment $X\in\{A,P,I\}$ on day *t*. It is defined as the proportion of traced contacts through compartment *X* were infected. It is related to the average transmission rate and the proportion of susceptible in the population. For compartment *A* and *P*, the average transmission rate is $\hat{\beta^{A}}=\beta^{A}$ and$\hat{\beta^{P}}=\beta^{P}$. For compartment *I*, the average transmission rate on day *t* is $\hat{\beta^{I}}(t)=\frac{\beta^{P}\left( \gamma^{P}+\pi^{P}\tau_{i}(t) \right)^{-1}+\beta^{I}\left( \gamma^{I}+\pi^{I}\tau_{i}(t) \right)^{-1}}{\left( \gamma^{P}+\pi^{P}\tau_{i}(t) \right)^{-1}+\left( \gamma^{I}+\pi^{I}\tau_{i}(t) \right)^{-1}}$. $\theta_{i,mn}^{E}\left( t \right)$ equals 0 since the individuals in *E* compartment are not infectious. Therefore, the infected contacts in city *i* in age group *m* who were infected and traced through infections in compartment $X\in\{E,A,P,I\}$ are removed at the rate of $\pi^{X}\tau_{i}(t)\kappa L\{\sum_{n=1}^{G} M_{mn}X_{i,n}\theta_{i,mn}^{X}\}$ on day *t.* And the infected contacts in city *i* in age group *m* are removed from compartment *Z* $\in\{E,A,P,I\}$ with the proportion of $p^{XZ}(t)$ on day *t*, which is $\pi^{X}\tau_{i}(t)\kappa L\{\sum_{n=1}^{G} M_{mn}X_{i,n}\theta_{i,mn}^{X}p_{i,n}^{XZ}\}$. $\sum_{Z\in\{E,A,P,I\}} p_{i,n}^{XZ}(t)=1$. $p_{i,n}^{XZ}(t)$ depends on dynamic of COVID-19 [4]. The contacts traced through the individuals in compartment *X* may be either not infected, or were infected by someone else rather than the identified cases. Therefore, these contacts are removed at the rate $\pi^{X}\tau_{i}(t)\kappa L\{\sum_{n=1}^{G} M_{mn}X_{i,n}(1-\theta_{i,mn}^{X})\}$ on day *t* and removed from compartment $Z^{'}\in\{S,V,E,A,P,I\}$ with the proportion of $Z_{i,m}^{'}/N_{i,m}$.

Other compartments and parameters were consistent with baseline model, where *R_0_* and *p_a_* was set according to the transmission dynamics parameters of Omicron, please refer to Tables S6 and S7. $\beta_{I}$ is calculated from the next generation matrix. We run 100 simulations to estimate the confidence interval for the disease burden.

In this model, the healthy individuals would go back to the susceptible compartment and may be infected later after the isolation. There are specialized centers/hospitals for isolation in China. These facilities may be overwhelmed if the COVID-19 outbreak is severe. The isolation at home would be preferred at this time. How the isolation is conducted is not modeled in our study. As for the testing capacity, it was extensively enlarged since 2022 in China. The regular testing was conducted in the cities with more than 10 million people. The testing facilities within a 15-minute walk of anywhere were provided in these cities. For other cities with less population, the regular testing was also conducted. In addition, the pooled PCR tests were employed to enlarge the capacity. The time from sample collection to returning the testing result was about 6h-12h. If the testing result is positive, a text message would be sent to the suspicious person and another PCR test was performed later to confirm the result. During this time, limited activities were suggested to lower the secondary transmission risk. Once getting the positive result, the SARS-CoV-2 infection would be isolated in specialized centers/hospitals. The specialized centers/hospitals were built to enlarge the isolation capacity.

**S2. A metapopulation model that incorporates the initial public health measures**

During the first COVID-19 wave in 2020 in China, a high-intensity nationwide travel restriction was used to control the Wuhan-Hu-1 wave, in which the number of recorded passenger inflows to all Chinese cities fell by 73.2%, on average (Figs S1–S4). The synchrony of COVID-19 dynamic between two cities declines with increasing geographical distance and decreasing travel movements (S4 Fig). Based on the fitted model in Section S1.2, the effectiveness of the initial public health measures against Wuhan-Hu-1 variant on the epidemic duration was evaluated by varying the strength of social distancing and the travel restriction between cities (S7A Fig). We also performed the similar analysis for Omicron-like variant by setting R_0_ to 10 (S7B Fig). The required intensity of social distancing for achieving suppression within a short period is insensitive to the changes in various levels of travel restriction between cities, implying a limited effect of travel restrictions between cites (S7B Fig). In the model of Section S1.2, the vaccinated individuals (*V_i_*) were incorporated. Using this compartment and the above fitted model, the effectiveness of COVID-19 vaccines with different coverages on the epidemic duration of Omicron-like wave is also assessed (S7C Fig). Because of lower vaccine efficacy in preventing infectiousness for Omicron, higher-intensity of social distancing and vaccine coverage are required to achieve suppression within 76 days, compared to Wuhan-Hu-1 variant (S7C Fig).

**S3. The burdens of hospitalizations and ICU admissions due to underlying health conditions**

The COVID-19 burden is calculated under the least stringent control strategy (i.e., 3-day-interval, response lag of 3 weeks) to get an estimation for a pessimistic scenario among all possible control strategies based on the stochastic age-stratified metapopulation model.

**S3.1 Baseline** **number of** **hospitalizations and ICU admissions by assuming** **all are healthy**

Inactivated vaccine (BBIBP-CorV and CoronaVac) distributed in China has protection against hospitalizations and ICU admissions. Total number of hospitalizations (${Hos}_{i, m}^{baseline}$) and ICU admissions (${ICU}_{i, m}^{baseline}$) in city *i* in age group *m* at baseline are therefore:

$${Hos}_{i, m}^{baseline}=p_{h,m}^{unvac}\left( 1-p_{a,m} \right){Case}_{i,m}^{unvac}+p_{h,m}^{vac}\left( 1-p_{a,m} \right){Case}_{i,m}^{vac}$$

$${ICU}_{i, m}^{baseline}=p_{h,m}^{unvac}p_{u,m}^{unvac}\left( 1-p_{a,m} \right){Case}_{i,m}^{unvac}+p_{h,m}^{vac}p_{u,m}^{vac}\left( 1-p_{a,m} \right){Case}_{i,m}^{vac}$$

${Case}_{m}^{unvac}$ is the total unvaccinated cases in city *i* in age group *m* and ${Case}_{m}^{vac}$ is the total vaccinated cases in city *i* in age group *m*. $p_{h,m}^{vac}$ and $p_{h,m}^{unvac}$ are the proportion of symptomatic infections requiring hospitalizations in age group *m* for vaccinated and unvaccinated individuals, and $p_{u,m}^{vac}$ and $p_{u,m}^{unvac}$ are the proportion of hospitalized patients requiring ICU in age group *m* for vaccinated and unvaccinated individuals. Note that ${Hos}_{i, m}^{baseline}$ is the total number of hospitalizations in city *i* in age group *m* at baseline level. $H_{i,m}^{baseline}\left( t \right)$ is the required number of hospitalizations in day *t* in city *i* and age group *m* at baseline level. The value of parameters are listed in S8 Table.

**S3.2 Number of hospitalizations and ICU admissions** **considering** **underlying health conditions**

Considering underlying health conditions of infected individuals, total number of hospitalizations (${Hos}_{i, m}^{d}$), and ICU admissions (${ICU}_{i, m}^{d}$) of infected individuals in city *i* in age group *m*, taking disease *d* into account, is calculated by:

$${Hos}_{i,m}^{d}={RR\_hos}^{d}{prv}_{i,m}^{d}{Hos}_{i, m}^{baseline}+(1-{prv}_{i,m}^{d}){Hos}_{i, m}^{baseline}$$

$${ICU}_{i,m}^{d}={RR\_icu}^{d}{prv}_{i,m}^{d}{ICU}_{i, m}^{baseline}+(1-{prv}_{i,m}^{d}){ICU}_{i, m}^{baseline}$$

$${prv}_{i,m}^{d}=min(\frac{{prv}_{nation,m}^{d}\times p_{nation,m}}{\sum_{m=1}^{G} {prv}_{nation,m}^{d}\times p_{nation,m}}\times\frac{{prv}_{i}^{d}}{p_{i,m}}, 1)$$

The hospitalizations and ICU admissions rates for Omicron infection of vaccinated and unvaccinated individuals with underlying disease *d* in age group *m* is calculated by the rates at baseline multiplied by the risk ratio of hospitalization (${RR\_hos}^{d}$), ICU admission (${RR\_icu}^{d}$) of the corresponding disease. Here, we considered the risk ratio is varaint-, and vaccine- independent. The prevalence of disease *d* in city *i* in age group *m* is dependent on the disease prevalence in age group *m* across nation (${prv}_{nation,m}^{d}$), overall prevalence in city *i* (${prv}_{i}^{d}$), the proportion of the age group *m* in the population of nation ($p_{nation,m}$), and the proportion of the age group *m* in population of city *i* ($p_{nation,m}$). We assumed the age distribution of prevalence of underlying health conditions is consistent between cities and country. Diseases with only age-specific (i.e.,chronic kidney disease) or city-specific (i.e., cancer and chronic obstructive pulmonary disease) prevalence data available are assumed to be homogeneous in terms of spatial distribution or age distribution. If the sum of ${prv}_{i,m}^{d}$ across all the diseases is larger than 1, ${prv}_{i,m}^{d}$ will be normalized to $\frac{{prv}_{i,m}^{d}}{\sum_{d=1}^{D} {prv}_{i,m}^{d}}$. Please refer to Table 1 for detailed data.

**S3.3 The calculation for the extra number of hospitalizations and ICU admissions due to underlying health conditions**

The total extra number of hospitalizations (${EHos}_{i,m}^{d}$) due to underlying health condition *d* is considered as the difference between the hospitalizations with underlying health condition *d* and the hospitalizations without underlying health condition *d*, and is given by:

$${EHos}_{i,m}^{d}= {Hos}_{i,m}^{d}-{Hos}_{i, m}^{baseline}$$

Similarly, the total extra number of ICU admissions (${EICU}_{i,m}^{d}$) due to underlying health condition *d* is given by:

$${EICU}_{i,m}^{d}={ICU}_{i,m}^{d}-{ICU}_{i, m}^{baseline}$$

**S3.4 The daily required hospital beds and ICU beds by accounting for underlying health conditions**

In section S1.3, the daily required hospital beds ($H_{i,m}^{baseline}\left( t \right)$) and ICU beds ($U_{i,m}^{baseline}\left( t \right)$) without considering the underlying health conditions were modelled. In this section, we modeled the daily required COVID-19 burden with underlying health conditions as follows:

$$H_{i,m}\left( t \right)=H_{i,m}^{baseline}\left( t \right)+\sum_{d=1}^{D} {(RR_{hos}}^{d}{prv}_{i,m}^{d}H_{i,m}^{baseline}\left( t \right)+\left( 1-{prv}_{i,m}^{d} \right)H_{i,m}^{baseline}\left( t \right)-H_{i,m}^{baseline}(t))$$

$$U_{i,m}\left( t \right)=U_{i,m}^{baseline}\left( t \right)+\sum_{d=1}^{D} {(RR_{icu}}^{d}{prv}_{i,m}^{d}U_{i,m}^{baseline}\left( t \right)+\left( 1-{prv}_{i,m}^{d} \right)U_{i,m}^{baseline}\left( t \right)-U_{i,m}^{baseline}(t))$$

**S4.** **The calculation procedures for the parameters**

**S4.1 Proportion of infections that are asymptomatic for Omicron variant**

We collected the age and the clinical endpoint (i.e., asymptomatic or symptomatic) for each infection with Omicron during the outbreak of Beijing in the Spring of 2022. For each age group, we calculated the asymptomatic proportion as the number of asymptomatic infections divided by the total infections in this age group.

**S4.2 Sensitivity of PCR tests**

The time-dependent diagnostic sensitivity curve of the RT-PCT test was obtained from [5]. The sensitivity for each compartment was calculated as the average of the sensitivity during the corresponding period based on the curve.

**S4.3 Age-dependent hospitalization and ICU admission rates for symptomatic Omicron- infection**

The age-dependent hospitalization and ICU admission rates for unvaccinated individuals were obtained from the study of [6]. These rates may be affected by the underlying health conditions. To alleviate the effects of underlying conditions, we corrected the age-specific risk of hospitalization and ICU admission for age group >60 using the OR adjusted by the underlying health conditions from [7] (OR for hospitalization: 1.53; OR for ICU admission: 1.67). Specifically, the rate for age group >60 is calculated as the rate for age group 40-59 multiplied by the corresponding OR. Here we assume that the effects of underlying health condition on the hospitalization and ICU admission rates for age group 40-59 should be negligible. For the vaccinated individuals, the age-dependent hospitalization and ICU admission rates are calculated as the corresponding rates for unvaccinated individuals multiplied by the reduction due to the COVID-19 vaccines (i.e., 1- vaccine effectiveness). These parameters are shown in S8 Table.

**Reference**

1. Aleta A, Martín-Corral D, Pastore y Piontti A, Ajelli M, Litvinova M, Chinazzi M, et al. Modelling the impact of testing, contact tracing and household quarantine on second waves of COVID-19. Nat Hum Behav. 2020 Aug 5;4(9):964–71.

2. Laine M. MCMC toolbox for Matlab, Software. 2020 Oct; Available from: doi:10.5281/ZENODO.4105825

3. Bjørnstad ON. Epidemics: models and data using R. Springer Nature; 2023.

4. Lunz D, Batt G, Ruess J. To quarantine, or not to quarantine: A theoretical framework for disease control via contact tracing. Epidemics. 2021 Mar;34:100428.

5. Wells CR, Townsend JP, Pandey A, Moghadas SM, Krieger G, Singer B, et al. Optimal COVID-19 quarantine and testing strategies. Nat Commun. 2021 Jan 7;12(1):356.

6. Cai J, Deng X, Yang J, Sun K, Liu H, Chen Z, et al. Modeling transmission of SARS-CoV-2 Omicron in China. Nat Med. 2022 Jul 10;28(7):1468–75.

7. Robilotti E V., Babady NE, Mead PA, Rolling T, Perez-Johnston R, Bernardes M, et al. Determinants of COVID-19 disease severity in patients with cancer. Nat Med. 2020 Aug 24;26(8):1218–23.

8. Bundgaard H, Bundgaard JS, Raaschou-Pedersen DET, von Buchwald C, Todsen T, Norsk JB, et al. Effectiveness of adding a mask recommendation to other public health measures to prevent sars-cov-2 infection in danish mask wearers a randomized controlled trial. Ann Intern Med. 2021 Mar;174(3):335–43.

9. Cerqueira-Silva T, Andrews JR, Boaventura VS, Ranzani OT, de Araújo Oliveira V, Paixão ES, et al. Effectiveness of CoronaVac, ChAdOx1 nCoV-19, BNT162b2, and Ad26.COV2.S among individuals with previous SARS-CoV-2 infection in Brazil: a test-negative, case-control study. Lancet Infect Dis. 2022 Jun;22(6):791–801.

10. McMenamin ME, Nealon J, Lin Y, Wong JY, Cheung JK, Lau EHY, et al. Vaccine effectiveness of one, two, and three doses of BNT162b2 and CoronaVac against COVID-19 in Hong Kong: a population-based observational study. Lancet Infect Dis. 2022 Oct;22(10):1435–43.

11. Ranzani OT, Hitchings MDT, de Melo RL, de França GVA, Fernandes C de FR, Lind ML, et al. Effectiveness of an inactivated Covid-19 vaccine with homologous and heterologous boosters against Omicron in Brazil. Nat Commun. 2022 Oct 6;13(1):5536.

12. Cheung PHH, Chan CP, Jin DY. Lessons learned from the fifth wave of COVID-19 in Hong Kong in early 2022. Emerg Microbes Infect. 2022;11(1):1072–8.

13. Chen X, Yan X, Sun K, Zheng N, Sun R, Zhou J, et al. Estimation of disease burden and clinical severity of COVID-19 caused by Omicron BA.2 in Shanghai, February-June 2022. Emerg Microbes Infect. 2022 Dec 31;11(1):2800–7.

14. Geweke JF. Evaluating the Accuracy of Sampling-Based Approaches to the Calculations of Posterior Moments. Bayesian Stat. 1992;4:641–9.

15. Tian H, Liu Y, Li Y, Wu CH, Chen B, Kraemer MUG, et al. An investigation of transmission control measures during the first 50 days of the COVID-19 epidemic in China. Science. 2020 May 8;368(6491):638–42.

16. Burki TK. Omicron variant and booster COVID-19 vaccines. Lancet Respir Med. 2022 Feb;10(2):e17.

17. Buitrago-Garcia D, Egli-Gany D, Counotte MJ, Hossmann S, Imeri H, Ipekci AM, et al. Occurrence and transmission potential of asymptomatic and presymptomatic SARS-CoV-2 infections: A living systematic review and meta-analysis. Ford N, editor. PLOS Med. 2020 Sep 22;17(9):e1003346.

18. He Z, Ren L, Yang J, Guo L, Feng L, Ma C, et al. Seroprevalence and humoral immune durability of anti-SARS-CoV-2 antibodies in Wuhan, China: a longitudinal, population-level, cross-sectional study. Lancet. 2021 Mar;397(10279):1075–84.

19. Li XN, Huang Y, Wang W, Jing QL, Zhang CH, Qin PZ, et al. Effectiveness of inactivated SARS-CoV-2 vaccines against the Delta variant infection in Guangzhou: a test-negative case–control real-world study. Emerg Microbes Infect. 2021 Jan 1;10(1):1751–9.

20. Hao X, Cheng S, Wu D, Wu T, Lin X, Wang C. Reconstruction of the full transmission dynamics of COVID-19 in Wuhan. Nature. 2020 Aug 20;584(7821):420–4.

21. Backer JA, Eggink D, Andeweg SP, Veldhuijzen IK, van Maarseveen N, Vermaas K, et al. Shorter serial intervals in SARS-CoV-2 cases with Omicron BA.1 variant compared with Delta variant, the Netherlands, 13 to 26 December 2021. Eurosurveillance. 2022 Feb 10;27(6):1–5.

22. Del Águila-Mejía J, Wallmann R, Calvo-Montes J, Rodríguez-Lozano J, Valle-Madrazo T, Aginagalde-Llorente A. Secondary Attack Rate, Transmission and Incubation Periods, and Serial Interval of SARS-CoV-2 Omicron Variant, Spain. Emerg Infect Dis. 2022 Jun;28(6):1224–8.

23. Saad-Roy CM, Wagner CE, Baker RE, Morris SE, Farrar J, Graham AL, et al. Immune life history, vaccination, and the dynamics of SARS-CoV-2 over the next 5 years. Science. 2020 Nov 13;370(6518):811–8.

24. Zhang J, Litvinova M, Wang W, Wang Y, Deng X, Chen X, et al. Evolving epidemiology and transmission dynamics of coronavirus disease 2019 outside Hubei province, China: a descriptive and modelling study. Lancet Infect Dis. 2020 Jul;20(7):793–802.

25. Sayampanathan AA, Heng CS, Pin PH, Pang J, Leong TY, Lee VJ. Infectivity of asymptomatic versus symptomatic COVID-19. Lancet. 2021 Jan;397(10269):93–4.

26. Keeling MJ, Hollingsworth TD, Read JM. Efficacy of contact tracing for the containment of the 2019 novel coronavirus (COVID-19). J Epidemiol Community Health. 2020 Jun;74:861–6.

27. Shao J, Fan R, Hu J, Zhang T, Lee C, Huang X, et al. Clinical Progression and Outcome of Hospitalized Patients Infected with SARS-CoV-2 Omicron Variant in Shanghai, China. Vaccines. 2022 Aug 28;10(9):1409.

28. Chen J, Qi T, Liu L, Ling Y, Qian Z, Li T, et al. Clinical progression of patients with COVID-19 in Shanghai, China. J Infect. 2020 May;80(5):e1–6.

29. Rees EM, Nightingale ES, Jafari Y, Waterlow NR, Clifford S, B. Pearson CA, et al. COVID-19 length of hospital stay: a systematic review and data synthesis. BMC Med. 2020 Dec 3;18(1):270.

30. Zhang J, Litvinova M, Liang Y, Wang Y, Wang W, Zhao S, et al. Changes in contact patterns shape the dynamics of the COVID-19 outbreak in China. Science. 2020 Jun 26;368(6498):1481–6.

31. Zhang J, Litvinova M, Liang Y, Zheng W, Shi H, Vespignani A, et al. The impact of relaxing interventions on human contact patterns and SARS-CoV-2 transmission in China. Sci Adv. 2021 May;7(19):eabe2584.
