# Supplementary material for: Marginal effects of public health measures and COVID-19 disease burden in China: A large-scale modelling study
Source: PLoS Comput Biol. 2023 Sep 18;19(9):e1011492. doi: 10.1371/journal.pcbi.1011492 (PMC10538769; doi:10.1371/journal.pcbi.1011492)
Supplement: S1 Fig — The shading from light to dark represents the value from low to high. The base layer of the map is provided by GADM (File link: https://gadm.org/download_country.html; License information: https://gadm.org/license.html). (DOCX) [file pcbi.1011492.s002.docx]

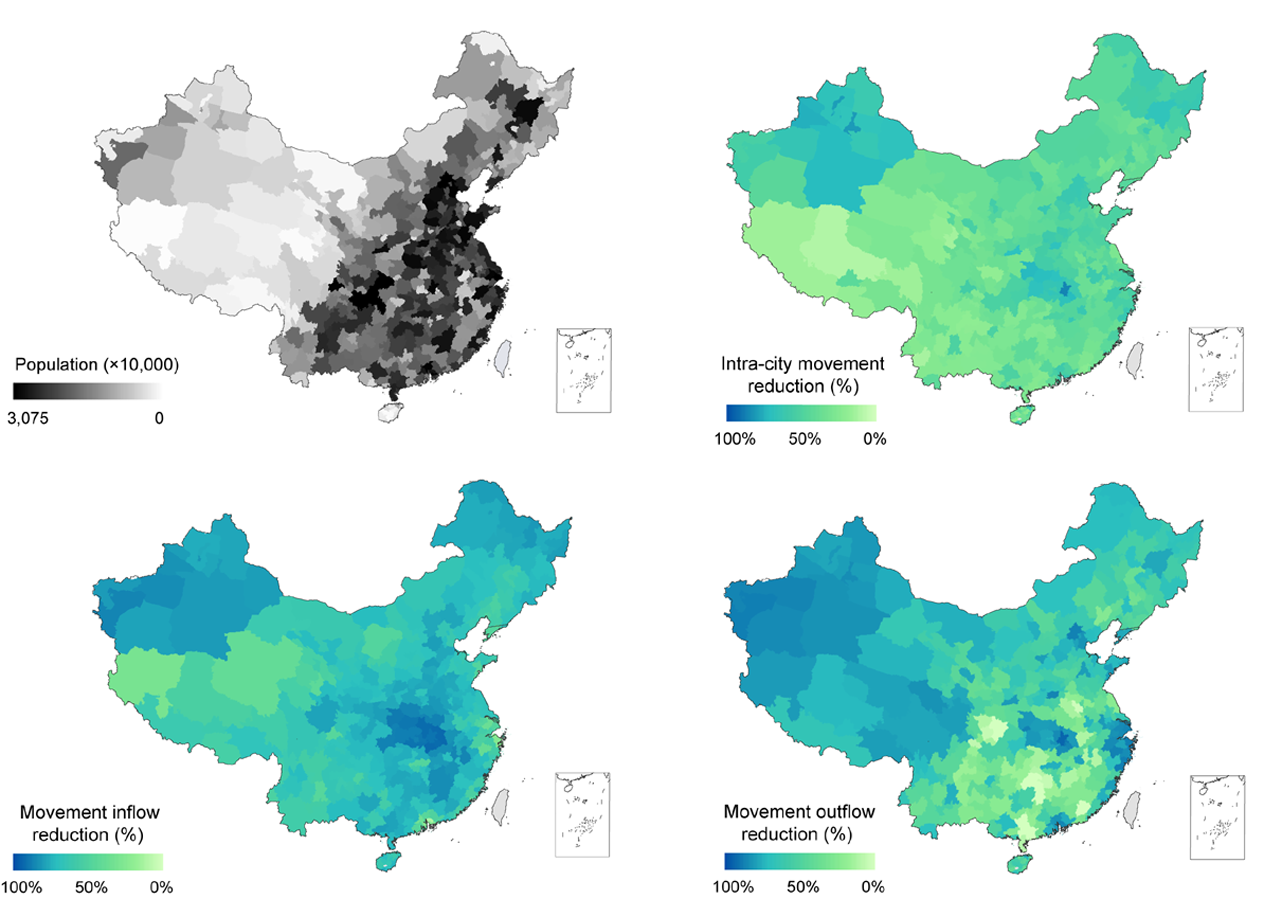


**Fig. S1. Heterogeneities in population distribution, intra-city movement reduction, movement inflow reduction, movement outflow reduction, before and after the travel restriction among Chinese cities during the first wave.** The shading from light to dark represents the value from low to high. The base layer of the map is provided by GADM (File link: https://gadm.org/download_country.html; License information: https://gadm.org/license.html).
