# Supplementary material for: Marginal effects of public health measures and COVID-19 disease burden in China: A large-scale modelling study
Source: PLoS Comput Biol. 2023 Sep 18;19(9):e1011492. doi: 10.1371/journal.pcbi.1011492 (PMC10538769; doi:10.1371/journal.pcbi.1011492)
Supplement: S2 Fig — (A) Movement inflows in 2019 and 2020, averaged across 366 Chinese cities. The median (solid line) and interquartile range (shading) of values among cities are shown. The vertical dotted line represents the beginning of the Wuhan lockdown on 23rd January 2020. (B) Intra-city movements in China before and after travel restriction during the SARS-CoV-2 pandemic between January 1 and March 6, 2020. Line shade indicates the average number of daily travel movements (>300 are shown) for each pair of location. Point size represents the volume of travel inflow. (DOCX) [file pcbi.1011492.s003.docx]

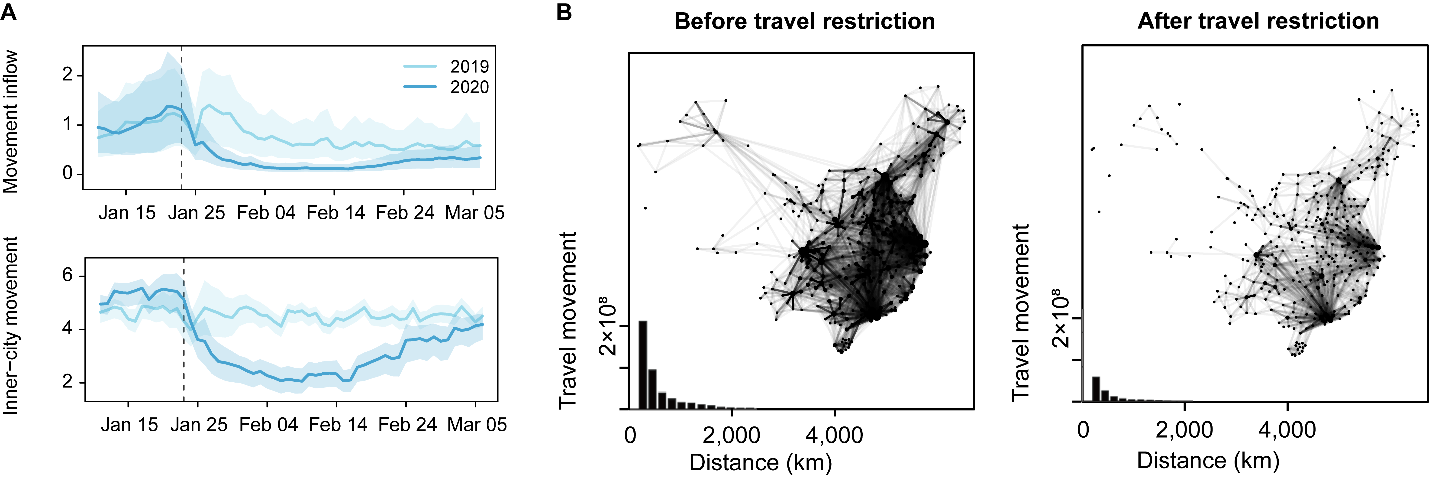


**Fig. S2. Travel movements in China before and after the travel restriction**. (**A**) Movement inflows in 2019 and 2020, averaged across 366 Chinese cities. The median (solid line) and interquartile range (shading) of values among cities are shown. The vertical dotted line represents the beginning of the Wuhan lockdown on 23rd January 2020. (**B**) Intra-city movements in China before and after travel restriction during the SARS-CoV-2 pandemic between January 1 and March 6, 2020. Line shade indicates the average number of daily travel movements (>300 are shown) for each pair of location. Point size represents the volume of travel inflow.
