# Supplementary material for: Marginal effects of public health measures and COVID-19 disease burden in China: A large-scale modelling study
Source: PLoS Comput Biol. 2023 Sep 18;19(9):e1011492. doi: 10.1371/journal.pcbi.1011492 (PMC10538769; doi:10.1371/journal.pcbi.1011492)
Supplement: S3 Fig — (A) Travel matrices before and after travel restriction. Lower-triangular values represent travel movements from city i to city j, and upper-triangular values from city j to city i. The shading from light to dark represents the volume of travel movements between pairs of cities from low to high. Black box represents the travel movements between cities within a province. The upper panel represents the total movements for each city, inflow (red) and outflow (blue). (B) Correlation of time series of reported cases between cities in the first wave of China. Cities are categorized by province and ranked from North (top) to South (bottom). (DOCX) [file pcbi.1011492.s004.docx]

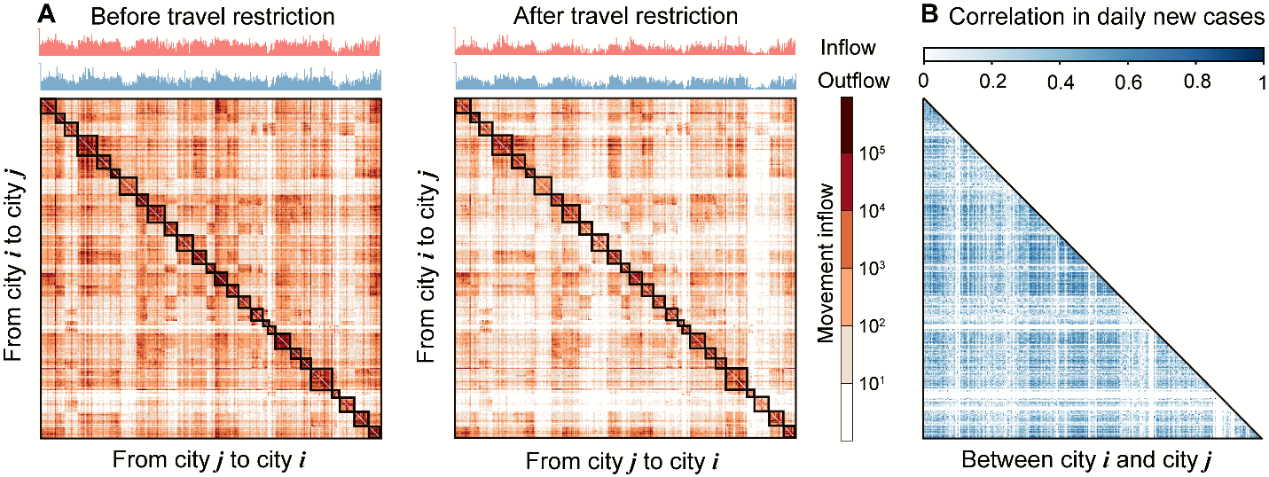


**Fig. S3. Travel movements and transmission pattern of the first SARS-CoV-2 wave in China.** (**A**) Travel matrices before and after travel restriction. Lower-triangular values represent travel movements from city *i* to city *j*, and upper-triangular values from city *j* to city *i*. The shading from light to dark represents the volume of travel movements between pairs of cities from low to high. Black box represents the travel movements between cities within a province. The upper panel represents the total movements for each city, inflow (red) and outflow (blue). (**B**) Correlation of time series of reported cases between cities in the first wave of China. Cities are categorized by province and ranked from North (top) to South (bottom).
