# Supplementary material for: Marginal effects of public health measures and COVID-19 disease burden in China: A large-scale modelling study
Source: PLoS Comput Biol. 2023 Sep 18;19(9):e1011492. doi: 10.1371/journal.pcbi.1011492 (PMC10538769; doi:10.1371/journal.pcbi.1011492)
Supplement: S4 Fig — (A) Each point represents a pair of cities. Synchrony of the epidemics in the two cities is measured by the correlation between the number of cases reported in two cities on each day, using a spatial non-parametric correlation function (black line = estimated curve, dark grey area 95% confidence band). The synchrony declines with increasing geographical distance. (B) Pairs of cities are ranked according to the level of travel movements between them (from low to high). The city pairs are then classified into ten categories corresponding to quantiles of the rank. Box and whisker plots show distribution of epidemic synchrony scores for cities in each of the ten categories; The first box represents the correlation among Q1-Q1 pairs, the second is among Q1-Q2 pairs, up to the final bar which is among Q4-Q4 pairs. Pairs of cities with more travel movements have more synchronized epidemics. (DOCX) [file pcbi.1011492.s005.docx]

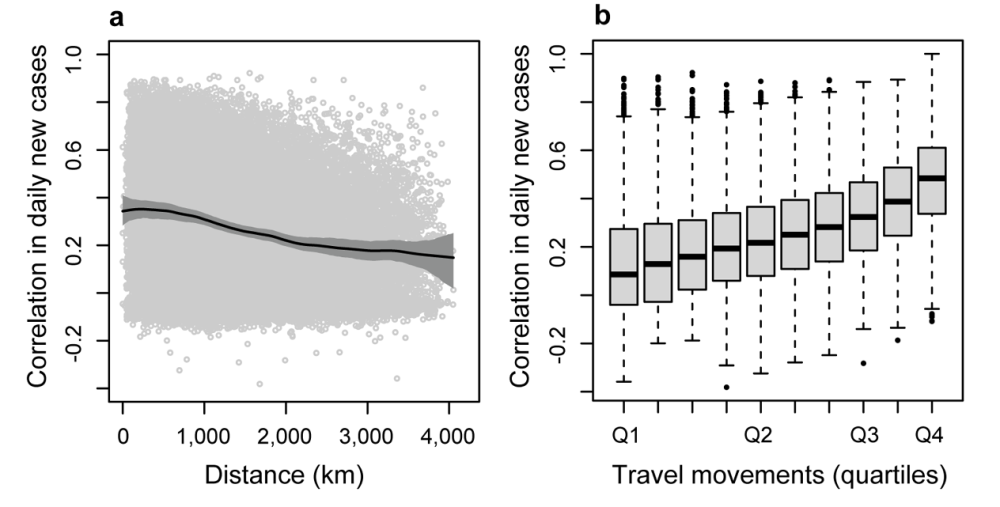


**Fig. S4. Pairwise correlation in daily COVID-19 cases between cities during the first wave in China.** (**A**) Each point represents a pair of cities. Synchrony of the epidemics in the two cities is measured by the correlation between the number of cases reported in two cities on each day, using a spatial non-parametric correlation function (black line = estimated curve, dark grey area 95% confidence band). The synchrony declines with increasing geographical distance. (**B**) Pairs of cities are ranked according to the level of travel movements between them (from low to high). The city pairs are then classified into ten categories corresponding to quantiles of the rank. Box and whisker plots show distribution of epidemic synchrony scores for cities in each of the ten categories; The first box represents the correlation among Q1-Q1 pairs, the second is among Q1-Q2 pairs, up to the final bar which is among Q4-Q4 pairs. Pairs of cities with more travel movements have more synchronized epidemics.
