# Supplementary material for: Marginal effects of public health measures and COVID-19 disease burden in China: A large-scale modelling study
Source: PLoS Comput Biol. 2023 Sep 18;19(9):e1011492. doi: 10.1371/journal.pcbi.1011492 (PMC10538769; doi:10.1371/journal.pcbi.1011492)
Supplement: S5 Fig — Correlation between the number of cases reported in each city and model fitting by March 6, 2020. Circle size is proportional to the correlation coefficient between time series of reported cases and model prediction for each city. (DOCX) [file pcbi.1011492.s006.docx]

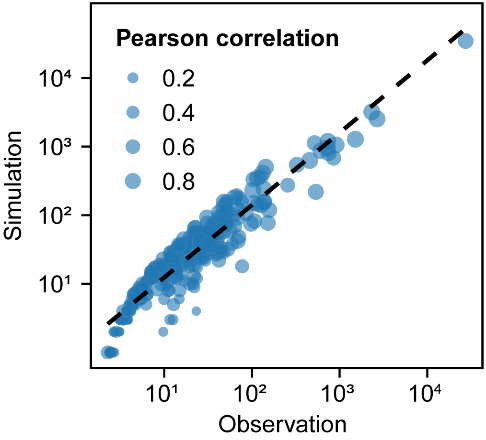


**Fig. S5. Fits of the meta-population model with the social distancing on transmission rate during the first wave in China**. Correlation between the number of cases reported in each city and model fitting by March 6, 2020. Circle size is proportional to the correlation coefficient between time series of reported cases and model prediction for each city.
