# Supplementary material for: Marginal effects of public health measures and COVID-19 disease burden in China: A large-scale modelling study
Source: PLoS Comput Biol. 2023 Sep 18;19(9):e1011492. doi: 10.1371/journal.pcbi.1011492 (PMC10538769; doi:10.1371/journal.pcbi.1011492)
Supplement: S6 Fig — The numbers of confirmed cases reported (points) and estimated (lines) each day in each city. Grey areas correspond to pointwise 95% prediction envelopes. (DOCX) [file pcbi.1011492.s007.docx]

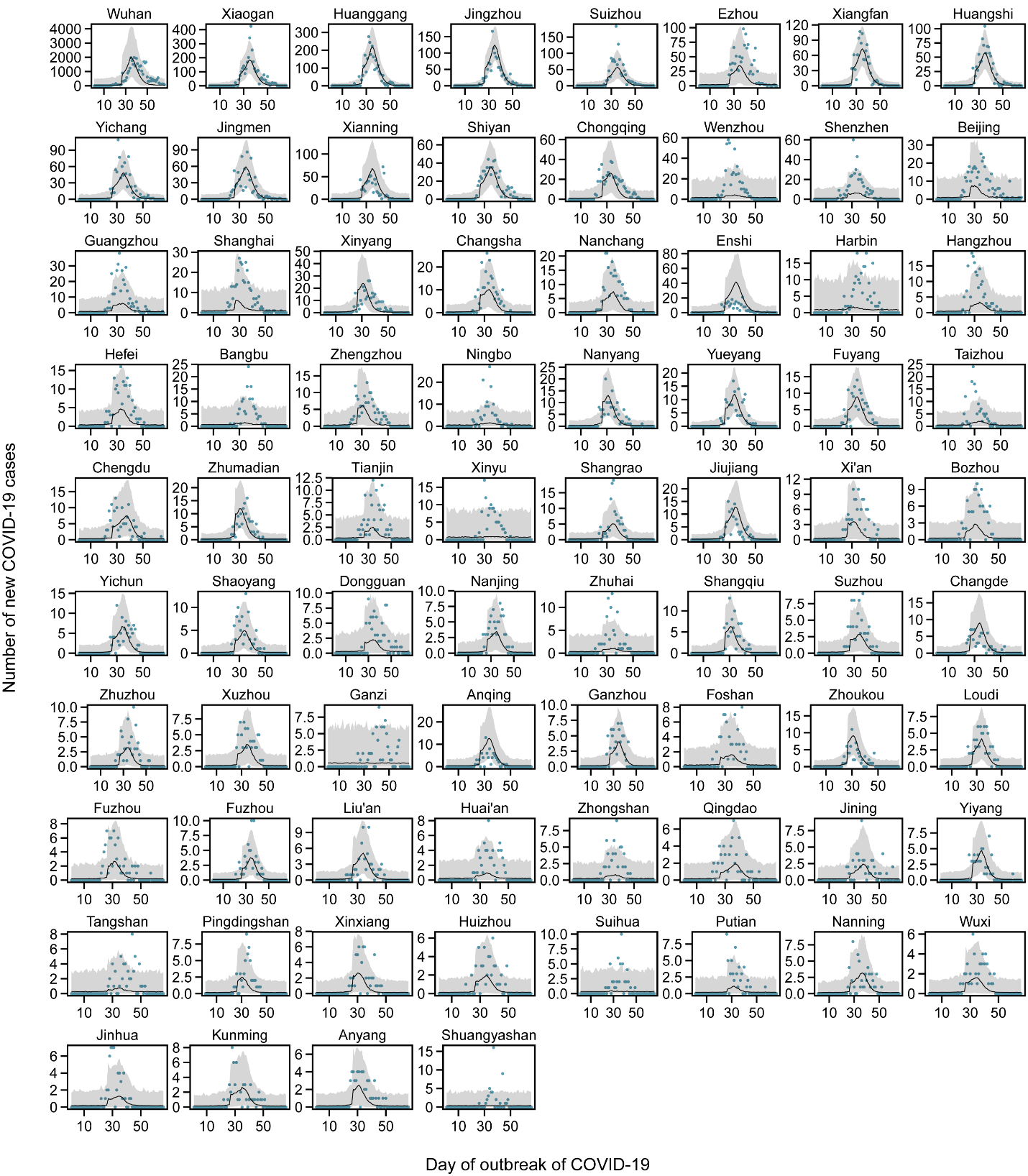


**Fig. S6. Fits of meta-population model with the social distancing on transmission rate to time series of reported cases from cities during the first wave (city with more than 50 cases are shown).** The numbers of confirmed cases reported (points) and estimated (lines) each day in each city. Grey areas correspond to pointwise 95% prediction envelopes.
