# Supplementary material for: Marginal effects of public health measures and COVID-19 disease burden in China: A large-scale modelling study
Source: PLoS Comput Biol. 2023 Sep 18;19(9):e1011492. doi: 10.1371/journal.pcbi.1011492 (PMC10538769; doi:10.1371/journal.pcbi.1011492)
Supplement: S7 Fig — (A) Predicted dependence of the average epidemic duration across all cities on travel restrictions between cities (from 100% strict restriction to 0% no restriction) and social distancing (a set of measures aiming at reduction of transmission rate, e.g., mask wearing, from strong [100%] to weak [0%]) for Wuhan-Hu-1 variant in the absence of vaccination. The estimated intensity of social distancing on reduction of transmission rate during the first wave in 2020 in China is indicated by the red cross. The fitting performance for the first wave can be found in S5 and S6 Figs. The simulation is based on the specific NPIs implemented during the first wave in 2020, except the strength of travel restrictions between cities. (B) Same as in (A) but for Omicron variant. (C) Predicted dependence of average epidemic duration across all Chinese cities on vaccine coverage and social distancing for the Omicron variant. The simulation is based on the specific NPIs implemented during the first wave in 2020. In (A) to (C), the Zero-COVID line (i.e., controlling the outbreak within 76 days) is shown as a dashed white line and solid line for Wuhan-Hu-1 and Omicron variants, respectively (see Methods). Note that the darker color with longer duration indicates that the whole population was infected, and the epidemic curve is either thin or flat. Effectiveness of China’s inactivated vaccines (BBIBP-CorV and CoronaVac) against infection was set to 40% for Omicron and 59% for Wuhan-Hu-1. The full list of epidemiological parameters is given in S6 Table. (DOCX) [file pcbi.1011492.s008.docx]

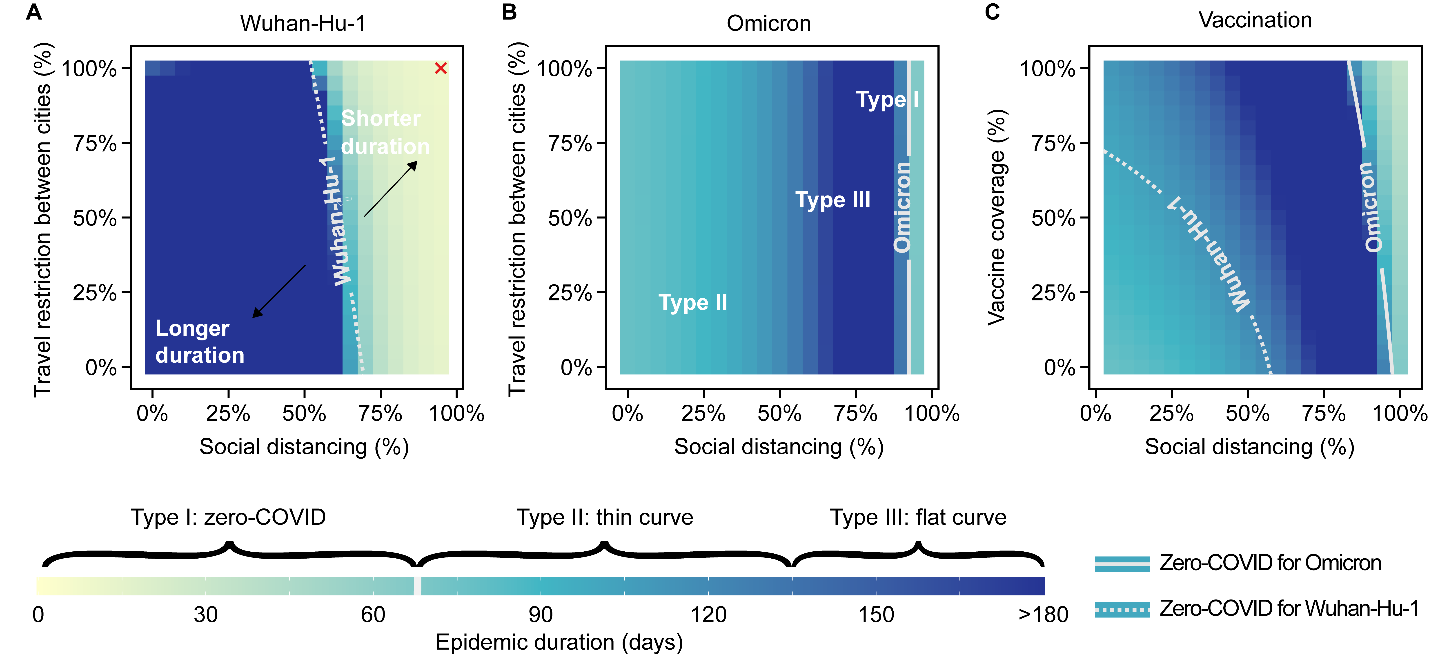


**Fig. S7. Effectiveness of public health measures without population-level testing during Wuhan-Hu-1 and Omicron waves in China**. (**A**) Predicted dependence of the average epidemic duration across all cities on travel restrictions between cities (from 100% strict restriction to 0% no restriction) and social distancing (a set of measures aiming at reduction of transmission rate, e.g., mask wearing, from strong [100%] to weak [0%]) for Wuhan-Hu-1 variant in the absence of vaccination. The estimated intensity of social distancing on reduction of transmission rate during the first wave in 2020 in China is indicated by the red cross. The fitting performance for the first wave can be found in Figs S5 and S6. The simulation is based on the specific NPIs implemented during the first wave in 2020, except the strength of travel restrictions between cities. (**B**) Same as in (A) but for Omicron variant. (**C**) Predicted dependence of average epidemic duration across all Chinese cities on vaccine coverage and social distancing for the Omicron variant. The simulation is based on the specific NPIs implemented during the first wave in 2020. In (A) to (C), the Zero-COVID line (i.e., controlling the outbreak within 76 days) is shown as a dashed white line and solid line for Wuhan-Hu-1 and Omicron variants, respectively (see Methods). Note that the darker color with longer duration indicates that the whole population was infected, and the epidemic curve is either thin or flat. Effectiveness of China’s inactivated vaccines (BBIBP-CorV and CoronaVac) against infection was set to 40% for Omicron and 59% for Wuhan-Hu-1. The full list of epidemiological parameters is given in S6 Table.
