# Supplementary material for: Marginal effects of public health measures and COVID-19 disease burden in China: A large-scale modelling study
Source: PLoS Comput Biol. 2023 Sep 18;19(9):e1011492. doi: 10.1371/journal.pcbi.1011492 (PMC10538769; doi:10.1371/journal.pcbi.1011492)
Supplement: S9 Fig — Predicted number of tests across 366 cities in China under different testing intervals and response lags. The grey dot represents the median and the grey error bar represents the 95% CI based on the 100 simulations. The reduction of social distancing on transmission rate is set to 18% by considering the effect of only mask wearing against SARS-CoV-2 infection [8]. The vaccine coverage is set to 89% (consistent with 86% vaccine coverage in the ≥60 age group by August of 2022 in China) and the effectiveness of China’s inactivated vaccine (BBIBP-CorV and CoronaVac) against infection was set to be 40% for Omicron [9]. Note that when response lag is 3 weeks, cities with small population size may suffer from lockdown due to high proportion of population of cities were traced, and the outbreak is quickly contained, resulting in smaller number of tests. (DOCX) [file pcbi.1011492.s010.docx]

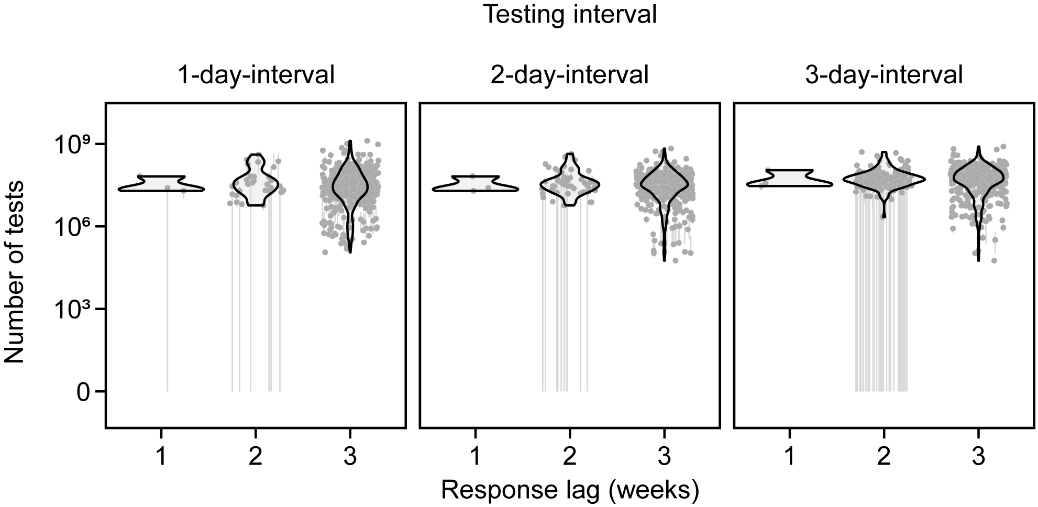


**Fig. S9. Number of tests under different response lags and testing intervals of population-level testing (*R*_0_=10)**. Predicted number of tests across 366 cities in China under different testing intervals and response lags. The grey dot represents the median and the grey error bar represents the 95% CI based on the 100 simulations. The reduction of social distancing on transmission rate is set to 18% by considering the effect of only mask wearing against SARS-CoV-2 infection [8]. The vaccine coverage is set to 89% (consistent with 86% vaccine coverage in the ≥60 age group by August of 2022 in China) and the effectiveness of China’s inactivated vaccine (BBIBP-CorV and CoronaVac) against infection was set to be 40% for Omicron [9]. Note that when response lag is 3 weeks, cities with small population size may suffer from lockdown due to high proportion of population of cities were traced, and the outbreak is quickly contained, resulting in smaller number of tests.
