# Supplementary material for: Marginal effects of public health measures and COVID-19 disease burden in China: A large-scale modelling study
Source: PLoS Comput Biol. 2023 Sep 18;19(9):e1011492. doi: 10.1371/journal.pcbi.1011492 (PMC10538769; doi:10.1371/journal.pcbi.1011492)
Supplement: S13 Fig — Predicted epidemic duration and rounds of testing across 366 cities in China under different testing intervals and response lags when travel restrictions between cities is implemented with population-level testing. The grey dot represents the median and the grey error bar represents the 95% CI based on the 100 simulations. The dashed red line shows the duration of Wuhan’s lockdown in 2020 (i.e., 76 days) as reference. The number of cities with local outbreaks is labeled below the violin plot. The reduction of social distancing on transmission rate is set to 18% by considering the effect of only mask wearing against SARS-CoV-2 infection. The vaccine coverage is set to 89% (consistent with 86% vaccine coverage in the ≥60 age group by August of 2022 in China) and the effectiveness of China’s inactivated vaccine (BBIBP-CorV and CoronaVac) against infection was set to be 40% for Omicron. (DOCX) [file pcbi.1011492.s014.docx]

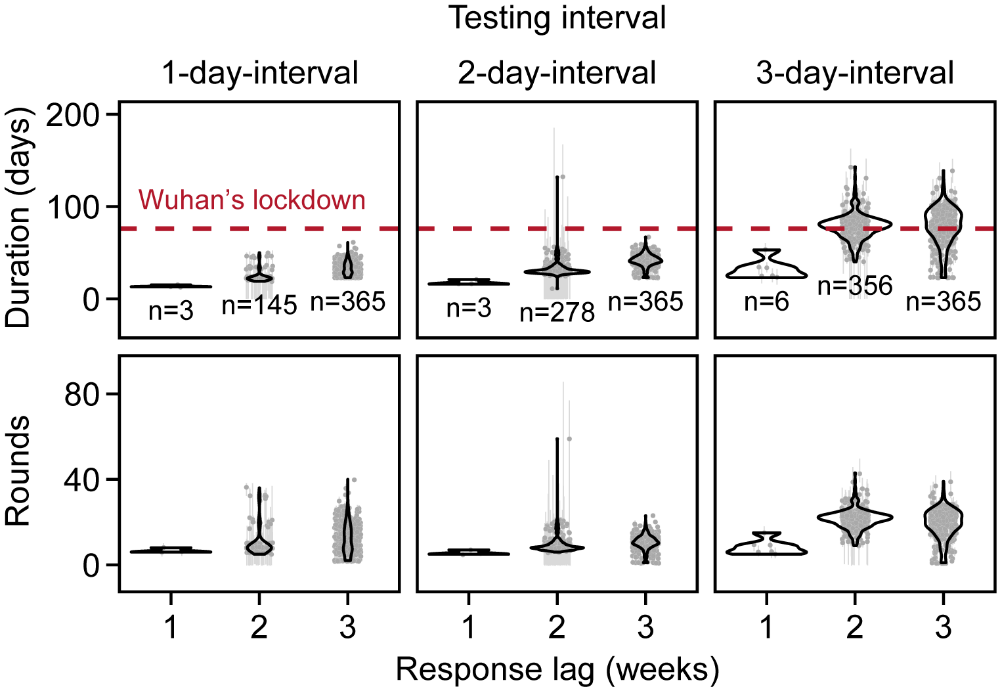


**Fig. S13.** **Effectiveness of population-level testing in China during Omicron wave (R_0_=10) when rI = 2.** Predicted epidemic duration and rounds of testing across 366 cities in China under different testing intervals and response lags when travel restrictions between cities is implemented with population-level testing. The grey dot represents the median and the grey error bar represents the 95% CI based on the 100 simulations. The dashed red line shows the duration of Wuhan’s lockdown in 2020 (i.e., 76 days) as reference. The number of cities with local outbreaks is labeled below the violin plot. The reduction of social distancing on transmission rate is set to 18% by considering the effect of only mask wearing against SARS-CoV-2 infection. The vaccine coverage is set to 89% (consistent with 86% vaccine coverage in the ≥60 age group by August of 2022 in China) and the effectiveness of China’s inactivated vaccine (BBIBP-CorV and CoronaVac) against infection was set to be 40% for Omicron.
