# Supplementary material for: Marginal effects of public health measures and COVID-19 disease burden in China: A large-scale modelling study
Source: PLoS Comput Biol. 2023 Sep 18;19(9):e1011492. doi: 10.1371/journal.pcbi.1011492 (PMC10538769; doi:10.1371/journal.pcbi.1011492)
Supplement: S14 Fig — Response lag is set to 2 weeks. A negative value corresponds to benefits yielded for duration or rounds by speeding up testing (shorten testing interval), and a positive value corresponds to potential loss for duration or rounds by speeding up testing. The red and blue dots represent median and grey error bar represents the 95% CI based on the 100 simulations. The reduction of social distancing on transmission rate is set to 18% by considering the effect of only mask wearing against SARS-CoV-2 infection [8]. The vaccine coverage is set to 89% (consistent with 86% vaccine coverage in the ≥60 age group by August of 2022 in China) and the effectiveness of China’s inactivated vaccine (BBIBP-CorV and CoronaVac) against infection was set to be 40% for Omicron [9]. (DOCX) [file pcbi.1011492.s015.docx]

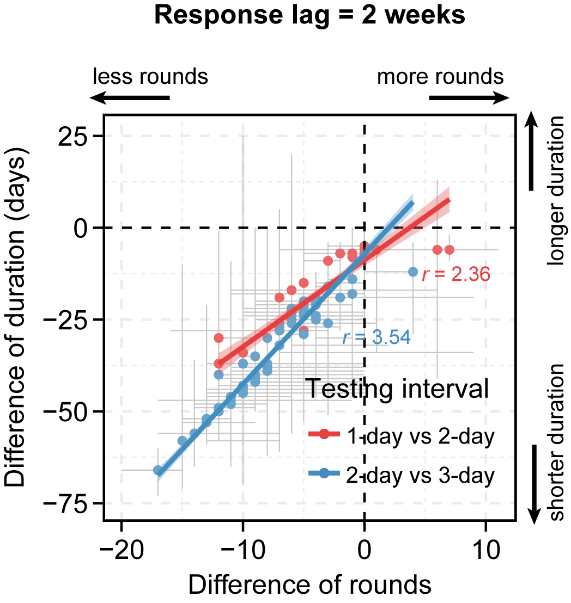


**Fig. S14.** **Comparison of epidemic duration and rounds of testing between two successive testing intervals without travel restrictions between cities (*R*_0_=10).** Response lag is set to 2 weeks. A negative value corresponds to benefits yielded for duration or rounds by speeding up testing (shorten testing interval), and a positive value corresponds to potential loss for duration or rounds by speeding up testing. The red and blue dots represent median and grey error bar represents the 95% CI based on the 100 simulations. The reduction of social distancing on transmission rate is set to 18% by considering the effect of only mask wearing against SARS-CoV-2 infection [8]. The vaccine coverage is set to 89% (consistent with 86% vaccine coverage in the ≥60 age group by August of 2022 in China) and the effectiveness of China’s inactivated vaccine (BBIBP-CorV and CoronaVac) against infection was set to be 40% for Omicron [9].
