# Supplementary material for: Marginal effects of public health measures and COVID-19 disease burden in China: A large-scale modelling study
Source: PLoS Comput Biol. 2023 Sep 18;19(9):e1011492. doi: 10.1371/journal.pcbi.1011492 (PMC10538769; doi:10.1371/journal.pcbi.1011492)
Supplement: S16 Fig — (A) The total SARS-CoV-2 infections for different age groups when testing interval is 3 days and response lag is 3 weeks. The dot represents the infections for an age group and a city. (B) Association between population size and proportion of isolated population across 366 cities in China when testing interval is 3 days and response lag is 3 weeks. The size and color of the circle represent the movement flow and the proportion of isolated population in a given city, respectively. (DOCX) [file pcbi.1011492.s017.docx]

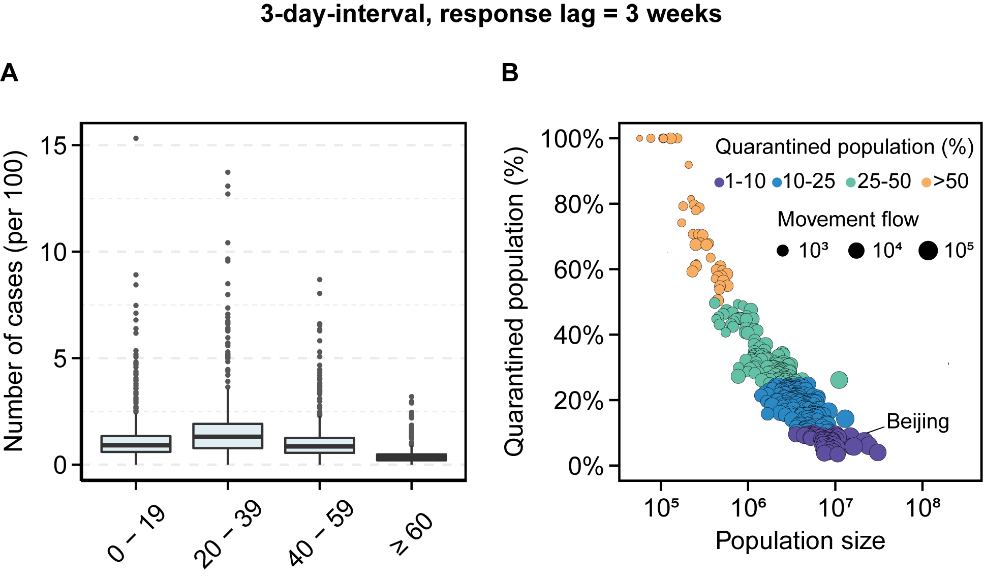


**Fig. S16.** **Potential impact of control strategy with long response lag on SARS-CoV-2 infections and daily life**. (**A**) The total SARS-CoV-2 infections for different age groups when testing interval is 3 days and response lag is 3 weeks. The dot represents the infections for an age group and a city. (**B**) Association between population size and proportion of isolated population across 366 cities in China when testing interval is 3 days and response lag is 3 weeks. The size and color of the circle represent the movement flow and the proportion of isolated population in a given city, respectively.
