# Supplementary material for: Marginal effects of public health measures and COVID-19 disease burden in China: A large-scale modelling study
Source: PLoS Comput Biol. 2023 Sep 18;19(9):e1011492. doi: 10.1371/journal.pcbi.1011492 (PMC10538769; doi:10.1371/journal.pcbi.1011492)
Supplement: S17 Fig — (A) Daily required hospital beds for different age groups. (B) Daily required ICU beds for different age groups. The response lag is set to be 3 weeks. The grey error bar or shadow represents the 95% CI for 100 simulations. The red dashed line represents the total available hospital beds or ICU beds in China. The vaccine coverage for all age groups was set to be 89%, consistent with 86% vaccine coverage in the ≥60 age group by August of 2022 in China. (DOCX) [file pcbi.1011492.s018.docx]

**
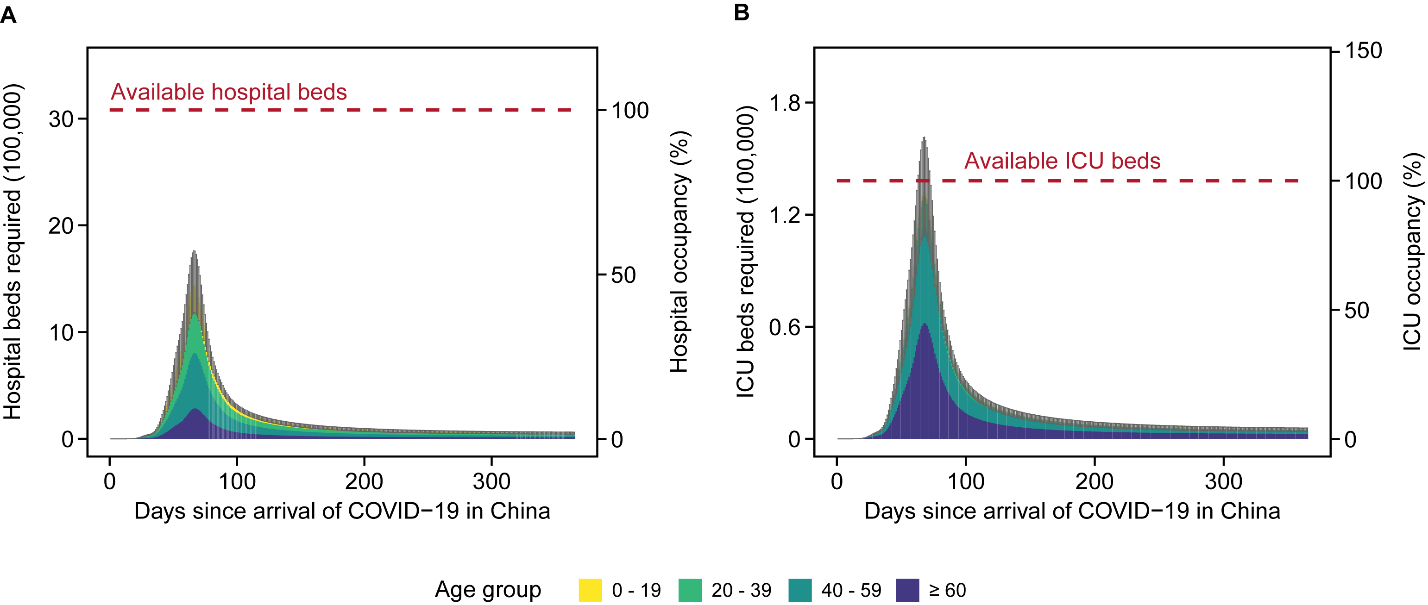
**

**Fig. S17. COVID-19 burden for Omicron-like variant (*R*_0_ = 10) under 4-day testing interval.** (**A**) Daily required hospital beds for different age groups. (**B**) Daily required ICU beds for different age groups. The response lag is set to be 3 weeks. The grey error bar or shadow represents the 95% CI for 100 simulations. The red dashed line represents the total available hospital beds or ICU beds in China. The vaccine coverage for all age groups was set to be 89%, consistent with 86% vaccine coverage in the ≥60 age group by August of 2022 in China.
