# Supplementary material for: Marginal effects of public health measures and COVID-19 disease burden in China: A large-scale modelling study
Source: PLoS Comput Biol. 2023 Sep 18;19(9):e1011492. doi: 10.1371/journal.pcbi.1011492 (PMC10538769; doi:10.1371/journal.pcbi.1011492)
Supplement: S18 Fig — (A) Daily required hospital beds for different age groups. (B) Daily required ICU beds for different age groups. The control strategy was employed with a testing interval of 3 days and a response lag of 3 weeks (the effective reproduction number Re < 1), which is the least stringent strategy aiming at suppressing SARS-CoV-2, as shown in Fig 2A. A strategy with a testing interval of 4 days and a response lag of 3 weeks would lead to the endemic of COVID-19 as shown in the grey dashed line with Re ≈ 1. The red dashed line represents the total available hospital beds or ICU beds in China. The grey solid line represents the peak number of required hospital beds or ICU beds during the pandemic without testing (Re > 1). The grey error bar or shadow represents the 95% CI for 100 simulations. The vaccine coverage for all age groups was set to be 89%, consistent with 86% vaccine coverage in the ≥60 age group by August of 2022 in China. The effectiveness of China’s inactivated vaccine (BBIBP-CorV and CoronaVac) against hospitalization and ICU admission were set to be 78.8% considering an optimistic scenario [10,11]. (DOCX) [file pcbi.1011492.s019.docx]

*
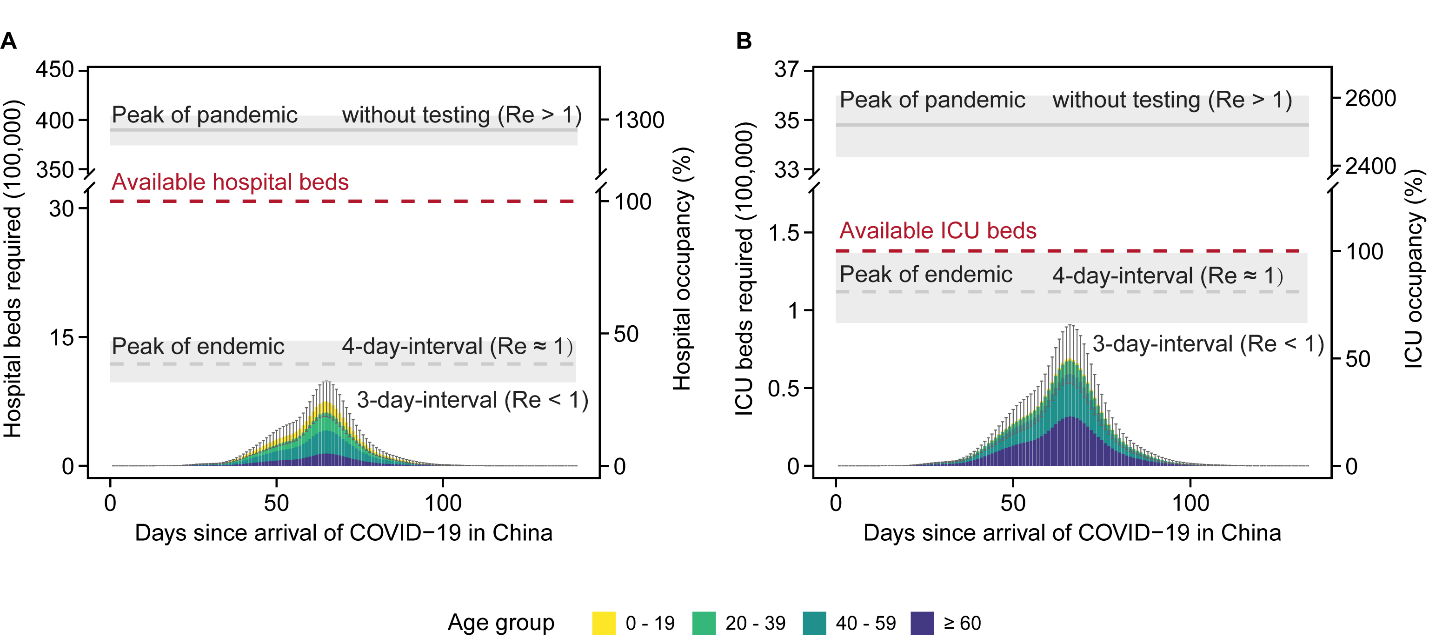
*

**Fig. S18.** **COVID-19 burden for Omicron-like variant (R_0_ = 10) under the control strategy in China for optimistic scenario. (A)** Daily required hospital beds for different age groups. (B) Daily required ICU beds for different age groups. The control strategy was employed with a testing interval of 3 days and a response lag of 3 weeks (the effective reproduction number R_e_ < 1), which is the least stringent strategy aiming at suppressing SARS-CoV-2, as shown in Figure 2A. A strategy with a testing interval of 4 days and a response lag of 3 weeks would lead to the endemic of COVID-19 as shown in the grey dashed line with R_e_ ≈ 1. The red dashed line represents the total available hospital beds or ICU beds in China. The grey solid line represents the peak number of required hospital beds or ICU beds during the pandemic without testing (R_e_ > 1). The grey error bar or shadow represents the 95% CI for 100 simulations. The vaccine coverage for all age groups was set to be 89%, consistent with 86% vaccine coverage in the ≥60 age group by August of 2022 in China. The effectiveness of China’s inactivated vaccine (BBIBP-CorV and CoronaVac) against hospitalization and ICU admission were set to be 78.8% considering an optimistic scenario [10,11].
