# Supplementary material for: Marginal effects of public health measures and COVID-19 disease burden in China: A large-scale modelling study
Source: PLoS Comput Biol. 2023 Sep 18;19(9):e1011492. doi: 10.1371/journal.pcbi.1011492 (PMC10538769; doi:10.1371/journal.pcbi.1011492)
Supplement: S20 Fig — (A) Daily required hospital beds for different age groups. (B) Daily required ICU beds for different age groups. The control strategy was employed with a testing interval of 3 days and a response lag of 3 weeks (the effective reproduction number Re < 1), which is the least stringent strategy aiming at suppressing SARS-CoV-2, as shown in Fig 2A. A strategy with a testing interval of 4 days and a response lag of 3 weeks would lead to the endemic of COVID-19 as shown in the grey dashed line with Re ≈ 1. The red dashed line represents the total available hospital beds or ICU beds in China. The grey dotted line represents the strategy with a testing interval of 4 days and a response lag of 3 weeks. The grey solid line represents the peak number of required hospital beds or ICU beds during the pandemic without testing (Re > 1). The grey error bar or shadow represents the 95% CI for 100 simulations. The vaccine coverage for all age groups was set to be 89%, consistent with 86% vaccine coverage in the ≥60 age group by August of 2022 in China. The age-dependent hospitalization and ICU admission rates were set according to data from the fifth wave of COVID-19 in Hong Kong in early 2022 [12]. (DOCX) [file pcbi.1011492.s021.docx]

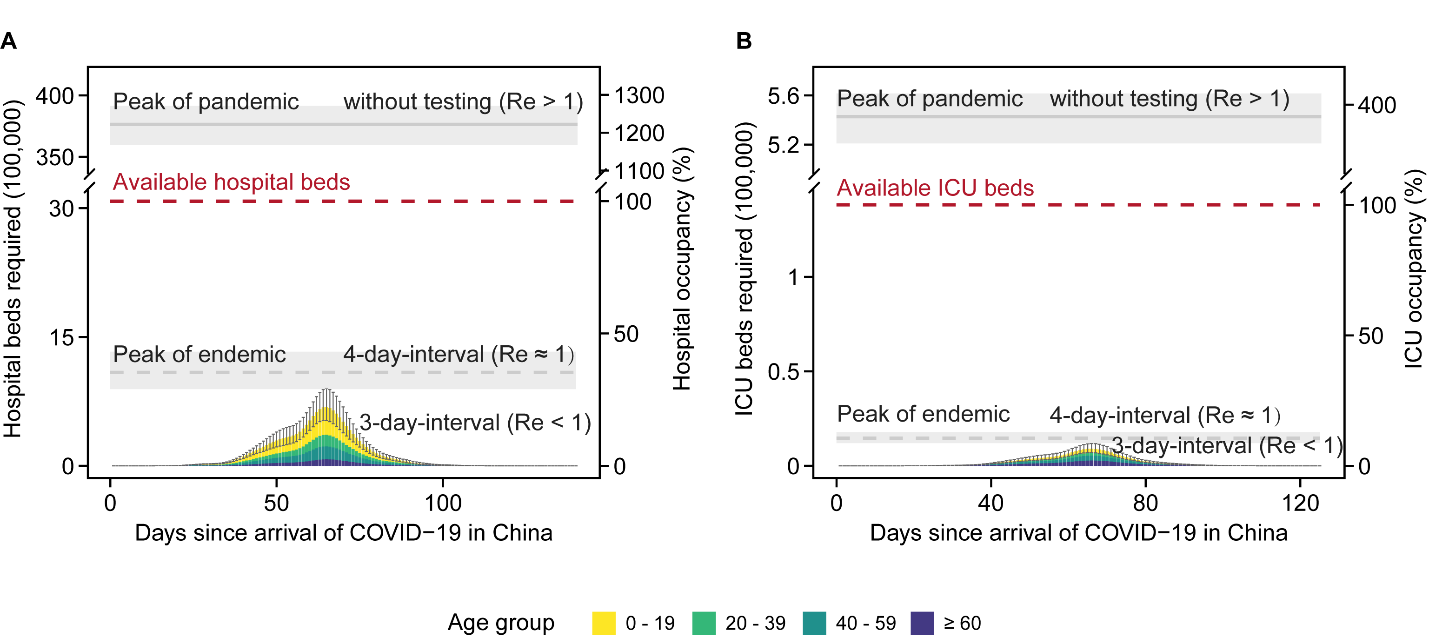


**Fig. S20.** **COVID-19 burden for Omicron-like variant (R_0_ = 10) under the control strategy in China for Hong Kong-specific age-dependent hospitalization and ICU admission rates. (A)** Daily required hospital beds for different age groups. (B) Daily required ICU beds for different age groups. The control strategy was employed with a testing interval of 3 days and a response lag of 3 weeks (the effective reproduction number R_e_ < 1), which is the least stringent strategy aiming at suppressing SARS-CoV-2, as shown in Figure 2A. A strategy with a testing interval of 4 days and a response lag of 3 weeks would lead to the endemic of COVID-19 as shown in the grey dashed line with R_e_ ≈ 1. The red dashed line represents the total available hospital beds or ICU beds in China. The grey dotted line represents the strategy with a testing interval of 4 days and a response lag of 3 weeks. The grey solid line represents the peak number of required hospital beds or ICU beds during the pandemic without testing (R_e_ > 1). The grey error bar or shadow represents the 95% CI for 100 simulations. The vaccine coverage for all age groups was set to be 89%, consistent with 86% vaccine coverage in the ≥60 age group by August of 2022 in China. The age-dependent hospitalization and ICU admission rates were set according to data from the fifth wave of COVID-19 in Hong Kong in early 2022 [12].
