# Supplementary material for: Marginal effects of public health measures and COVID-19 disease burden in China: A large-scale modelling study
Source: PLoS Comput Biol. 2023 Sep 18;19(9):e1011492. doi: 10.1371/journal.pcbi.1011492 (PMC10538769; doi:10.1371/journal.pcbi.1011492)
Supplement: S23 Fig — (A) The number of hospitalizations and (B) ICU admissions when the least stringent control strategy is employed. We considered all the infected individuals were healthy in the baseline scenario. The grey error bar or shadow represents the 95% CI for 100 simulations. (DOCX) [file pcbi.1011492.s024.docx]

*
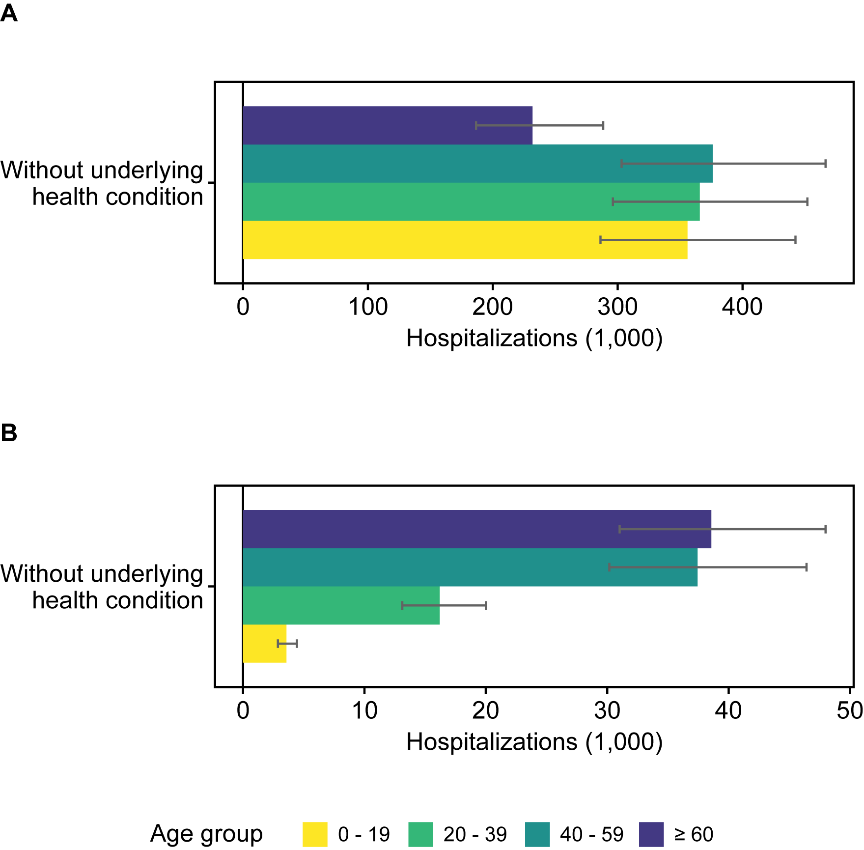
*

**Fig. S23. The disease burden for Omicron-like variant (*R*_0_=10) outbreak without considering the underlying health conditions.** (**A**) The number of hospitalizations and (**B**) ICU admissions when the least stringent control strategy is employed. We considered all the infected individuals were healthy in the baseline scenario. The grey error bar or shadow represents the 95% CI for 100 simulations.
