# Supplementary material for: Marginal effects of public health measures and COVID-19 disease burden in China: A large-scale modelling study
Source: PLoS Comput Biol. 2023 Sep 18;19(9):e1011492. doi: 10.1371/journal.pcbi.1011492 (PMC10538769; doi:10.1371/journal.pcbi.1011492)
Supplement: S1 Table — (DOCX) [file pcbi.1011492.s025.docx]

**Table S1**. The outbreaks controlled by public health measures in China.

| **City** | **Total cases** | **Epidemic duration (days)** | **Rounds of population-level testing** | **Response lag (days)** | **SARS-CoV-2 lineage** |
| --- | --- | --- | --- | --- | --- |
| **Xingtai** | 71 | 25 | 10 | 3 | B.1.1 |
| **Changchun** | 105 | 30 | 5 | 1 | B.1.1 |
| **Tonghua** | 307 | 44 | 3 | 8 | B.1.1 |
| **Shijiazhuang** | 869 | 24 | 7 | 4 | B.1.1 |
| **Nanjing** | 235 | 24 | 7 | 1 | Delta |
| **Yangzhou** | 570 | 30 | 23 | 0 | Delta |
| **Xiamen** | 236 | 21 | 10 | 2 | Delta |
| **Lanzhou** | 83 | 23 | 6 | 2 | Delta |
| **Dalian** | 308 | 24 | 5 | 3 | Delta |
| **Manzhouli** | 516 | 21 | 15 | 1 | Delta |
| **Shaoxing** | 387 | 21 | 6 | 2 | Delta |
| **Ningbo** | 74 | 13 | 5 | 1 | Delta |
| **Xian** | 2052 | 43 | 13 | 9 | Delta |
| **Zhengzhou** | 139 | 17 | 10 | 2 | Delta |
| **Xuchang** | 365 | 18 | 17 | 0 | Delta |
| **Anyang** | 467 | 22 | 11 | 2 | Omicron |
| **Tianjin** | 425 | 36 | 6 | 1 | Omicron |
| **Shenzhen** | 707 | 19 | 6 | 0 | Omicron |
