# Supplementary material for: Marginal effects of public health measures and COVID-19 disease burden in China: A large-scale modelling study
Source: PLoS Comput Biol. 2023 Sep 18;19(9):e1011492. doi: 10.1371/journal.pcbi.1011492 (PMC10538769; doi:10.1371/journal.pcbi.1011492)
Supplement: S2 Table — (DOCX) [file pcbi.1011492.s026.docx]

**Table S2**. Estimated parameters values in the first wave of 2020 in China.

| Parameter | Symbol | Mean | Std | *p*-value from geweke^*^ |  |
| --- | --- | --- | --- | --- | --- |
| Effect of social distancing on the reduction of transmission rate | *β_C_* | 0.99 | 0.00 | 0.99 |  |
| Detecting rate of symptomatic individuals in cities of China (except Hubei) before national emergency response | *δ* | 0.027 | 0.002 | 0.97 |  |
| Detecting rate of symptomatic individuals in cities of China (except Hubei) after national emergency response | *δ‘* | 0.157 | 0.006 | 0.97 |  |
| Detecting rate of symptomatic individuals in Wuhan city before national emergency response | *δ_Wh_* | 0.003 | 0.002 | 0.81 |  |
| Detecting rate of symptomatic individuals in Wuhan city after national emergency response | *δ_Wh_’* | 0.028 | 0.004 | 0.99 |  |
| Constant ratio between the detection rates of other cities in Hubei province and cities in China (excluding Hubei) after national emergency response | *δ_Hb_’* | 0.591 | 0.033 | 0.99 |  |
| The initial value for latent | *E_0_* | 1906.50 | 803.87 | 0.90 |  |
| The initial value for asymptomatic | *A_0_* | 2574.44 | 511.63 | 0.97 |  |
| The initial value for pre-symptomatic | *P_0_* | 549.61 | 444.51 | 0.85 |  |
| The initial value for infectious symptomatic | *I_0_* | 781.60 | 453.55 | 0.97 ­ |  |
| *geweke: a convergence diagnostic for Markov chains [14] | | | | | |
