# Supplementary material for: Marginal effects of public health measures and COVID-19 disease burden in China: A large-scale modelling study
Source: PLoS Comput Biol. 2023 Sep 18;19(9):e1011492. doi: 10.1371/journal.pcbi.1011492 (PMC10538769; doi:10.1371/journal.pcbi.1011492)
Supplement: S3 Table — (DOCX) [file pcbi.1011492.s027.docx]

**Table S3**. The significance of reduction in epidemic duration and rounds of testing by shorter response lag (weeks) from the t-test.

|  | **Epidemic duration** | | **Rounds of testing** | |
| --- | --- | --- | --- | --- |
| **Testing interval** | **Response lag: 1 vs 2 weeks** | **Response lag: 2 vs 3 weeks** | **Response lag: 1 vs 2 weeks** | **Response lag: 2 vs 3 weeks** |
| **1-day** | *P* <0.0001 | *P* <0.0001 | *P* <0.0001 | *P* <0.0001 |
| **2-day** | *P* <0.0001 | *P* <0.0001 | *P* <0.0001 | *P* <0.0001 |
| **3-day** | *P* <0.0001 | *P* <0.0001 | *P* <0.0001 | *P* <0.0001 |
