# Supplementary material for: Marginal effects of public health measures and COVID-19 disease burden in China: A large-scale modelling study
Source: PLoS Comput Biol. 2023 Sep 18;19(9):e1011492. doi: 10.1371/journal.pcbi.1011492 (PMC10538769; doi:10.1371/journal.pcbi.1011492)
Supplement: S4 Table — (DOCX) [file pcbi.1011492.s028.docx]

**Table S4**. The significance of reduction in epidemic duration and rounds of testing by shorter testing interval from the t-test.

|  | **Epidemic duration** | | **Rounds of testing** | |
| --- | --- | --- | --- | --- |
| **Response lag** | **1-day-interval vs 2-day-interval** | **2-day-interval vs 3-day-interval** | **1-day-interval vs 2-day-interval** | **2-day-interval vs 3-day-interval** |
| **1 weeks** | *P* =0.3622 | *P* =0.2459 | *P* =0.5000 | *P* =0.3024 |
| **2 weeks** | *P* <0.0001 | *P* <0.0001 | *P* =0.0045 | *P* <0.0001 |
| **3 weeks** | *P* <0.0001 | *P* <0.0001 | *P*=0.9841 | *P* <0.0001 |
