# Supplementary material for: Marginal effects of public health measures and COVID-19 disease burden in China: A large-scale modelling study
Source: PLoS Comput Biol. 2023 Sep 18;19(9):e1011492. doi: 10.1371/journal.pcbi.1011492 (PMC10538769; doi:10.1371/journal.pcbi.1011492)
Supplement: S5 Table — The isolated individuals include the individuals exposed to the SARS-CoV-2 and the infected individuals. (DOCX) [file pcbi.1011492.s029.docx]

**Table S5**. The number of cities with the peak number of isolated individuals reaching the population size during the outbreak under different combinations of testing intervals and response lags. The isolated individuals include the individuals exposed to the SARS-CoV-2 and the infected individuals.

| Response lag | 1-day-interval | 2-day-interval | 3-day-interval | 4-day-interval |
| --- | --- | --- | --- | --- |
| 1 week | 0 | 0 | 0 | 0 |
| 2 weeks | 0 | 0 | 0 | 0 |
| 3 weeks | 40 | 13 | 7 | 1 |
| 4 weeks | 335 | 196 | 132 | 97 |
