# Supplementary material for: Marginal effects of public health measures and COVID-19 disease burden in China: A large-scale modelling study
Source: PLoS Comput Biol. 2023 Sep 18;19(9):e1011492. doi: 10.1371/journal.pcbi.1011492 (PMC10538769; doi:10.1371/journal.pcbi.1011492)
Supplement: S6 Table — (DOCX) [file pcbi.1011492.s030.docx]

**Table S6**. Baseline model parameter values.

| Parameter | Symbol | Variant | Value | Data source |
| --- | --- | --- | --- | --- |
| Basic reproduction number | *R_0_* | Wuhan-Hu-1 | 3.2 | [15] |
|  |  | Omicron | 10 | [16] |
| Proportion of infections that are asymptomatic | *p_a_* | Wuhan-Hu-1 | 25% | [17,18]  Assumed |
|  |  | Omicron | 19%,  22%,  19%,  14% | Calculated based on COVID-19 outbreak in Beijing during the spring of 2022 |
| Effectiveness of China’s inactivated vaccine (BBIBP-CorV and CoronaVac) against symptomatic infection | *ε* | Wuhan-Hu-1 | 59% | [19] |
|  |  | Omicron | 40% | [9] |
| Latent period | 1/$\gamma^{E}$ | Wuhan-Hu-1 | 2.9 | [20] |
|  |  | Omicron | 1.2 | [6,21,22] |
| Pre-symptomatic period | 1/$\gamma^{P}$ | Wuhan-Hu-1 | 2.3 | [20] |
|  |  | Omicron | 2 | [6,21,22] |
| Mean infectious periods for asymptomatic individuals | 1/$\gamma^{A}$ | Wuhan-Hu-1 | 5 | [23,24] |
|  |  | Omicron | 3.5 | [21,22] |
| Mean infectious periods for symptomatic individuals | 1/$\gamma^{I}$ | Wuhan-Hu-1 | 5 | [23,24] |
|  |  | Omicron | 3.5 | [21,22] |
| Relative infectiousness of asymptomatic infections vs. symptomatic infections | $r_{1}$ |  | 1/3.84 | [25] |
| Relative infectiousness of pre-symptomatic infections vs. symptomatic infections | $r_{2}$ |  | 0.15 | [1] |
