# Supplementary material for: Marginal effects of public health measures and COVID-19 disease burden in China: A large-scale modelling study
Source: PLoS Comput Biol. 2023 Sep 18;19(9):e1011492. doi: 10.1371/journal.pcbi.1011492 (PMC10538769; doi:10.1371/journal.pcbi.1011492)
Supplement: S7 Table — (DOCX) [file pcbi.1011492.s031.docx]

**Table S7**. Population-level testing and contact tracing model parameter values.

| Parameter | Symbol | Value (range) | Data source |
| --- | --- | --- | --- |
| Social distancing (only wearing masks considered) on the reduction of transmission rate | $\beta_{C}$ | 18% | [8] |
| The fraction of contacts that can be  successfully traced | $\kappa$ | 0.17 | [26] |
| Average time from symptom onset to hospitalization | 1/$\gamma^{I2H}$ | 3.5 | [27,28] |
| Average time from symptom onset to ICU | 1/$\gamma^{I2U}$ | 3.5 | [27,28] |
| Length of hospital stay before recovery | 1/$\gamma^{H}$ | 6 | [6] |
| Length of ICU stay before recovery | 1/$\gamma^{U}$ | 8 | [29] |
| Sensitivity of PCR tests for the individuals in compartment E | $\pi^{E}$ | 0.41 | Calculated based on [5] |
| Sensitivity of PCR tests for the individuals in compartment A, P, I | $\pi^{A},\pi^{P},\pi^{I}$ | 0.94 | Calculated based on [5] |
| Population-level testing interval | $1/\tau$ | 1, 2, 3, 4 | Assumed |
| Contact tracing delay | *t*_0_ | 0 | Assumed |
| Duration of isolation | *1/q* | 14 | Assumed |
| Pre-defined contact tracing time  window | *L* | 14 | Assumed |
| Contacts matrix | *M* | - | [30,31] |
| The initial value for symptomatic | *I*_0_ | 50 | Assumed |
| The initial value for infectious asymptomatic | *A*_0_ | 50 | Assumed |
| The initial value for latent | *E*_0_ | 50 | Assumed |
| The initial value for pre-symptomatic | *P*_0_ | 50 | Assumed |
