# Supplementary material for: Marginal effects of public health measures and COVID-19 disease burden in China: A large-scale modelling study
Source: PLoS Comput Biol. 2023 Sep 18;19(9):e1011492. doi: 10.1371/journal.pcbi.1011492 (PMC10538769; doi:10.1371/journal.pcbi.1011492)
Supplement: S8 Table — The effectiveness of China’s inactivated vaccine (BBIBP-CorV and CoronaVac) against hospitalization and ICU admission were set to be 70% for Omicron for all age groups [11]. (DOCX) [file pcbi.1011492.s032.docx]

**Table S8**. Age-dependent hospitalization and ICU admission rates for symptomatic Omicron- infection in vaccinated and unvaccinated individuals. The effectiveness of China’s inactivated vaccine (BBIBP-CorV and CoronaVac) against hospitalization and ICU admission were set to be 70% for Omicron for all age groups [11].

| Clinical severity | Status | 0-19 | 20-39 | 40-59 | 60+ | Data source |
| --- | --- | --- | --- | --- | --- | --- |
| Hospitalization | Vaccinated ($p_{h,m}^{vac}$) | 0.03 | 0.03 | 0.04 | 0.06 | calculated |
|  | Unvaccinated ($p_{h,m}^{unvac}$) | 0.11 | 0.09 | 0.13 | 0.19 | [6] |
| ICU admission among hospitalized patients | Vaccinated ($p_{u,m}^{vac}$) | 0.01 | 0.02 | 0.05 | 0.09 | calculated |
|  | Unvaccinated ($p_{u,m}^{unvac}$) | 0.02 | 0.08 | 0.17 | 0.26 | [6] |
